# Analysis of a non-integer order mathematical model for double strains of dengue and COVID-19 co-circulation using an efficient finite-difference method

**DOI:** 10.1038/s41598-023-44825-w

**Published:** 2023-10-18

**Authors:** Emeka F. Obiajulu, Andrew Omame, Simeon C. Inyama, Uchenna H. Diala, Salman A. AlQahtani, Mabrook S. Al-Rakhami, Abdulaziz M. Alawwad, Abdullilah A. Alotaibi

**Affiliations:** 1 Department of Mathematics, Nnamdi Azikiwe University, P.O. Box 5025, Awka, 420110 Nigeria; 2 Department of Mathematics, Federal University of Technology, P.O. Box 1526, Owerri, 460114 Nigeria; 3 Department of Electrical and Electronic Engineering, School of Computing and Engineering, College of Science and Engineering, University of Derby, Derby, DE22 3AW UK; 4 New Emerging Technologies and 5G Network and Beyond Research Chair, Department of Computer Engineering, College of Computer and Information Sciences, King Saud University, P.O. Box 51178, Riyadh, 11543 Saudi Arabia; 5 Department of Information Systems, College of Computer and Information Sciences, King Saud University, P.O. Box 51178, Riyadh, 11543 Saudi Arabia; 6 Department of Computer Engineering, College of Computer and Information Sciences, King Saud University, P.O. Box 51178, Riyadh, 11543 Saudi Arabia

**Keywords:** Mathematics and computing, Applied mathematics

## Abstract

An efficient finite difference approach is adopted to analyze the solution of a novel fractional-order mathematical model to control the co-circulation of double strains of dengue and COVID-19. The model is primarily built on a non-integer Caputo fractional derivative. The famous fixed-point theorem developed by Banach is employed to ensure that the solution of the formulated model exists and is ultimately unique. The model is examined for stability around the infection-free equilibrium point analysis, and it was observed that it is stable (asymptotically) when the maximum reproduction number is strictly below unity. Furthermore, global stability analysis of the disease-present equilibrium is conducted via the direct Lyapunov method. The non-standard finite difference (NSFD) approach is adopted to solve the formulated model. Furthermore, numerical experiments on the model reveal that the trajectories of the infected compartments converge to the disease-present equilibrium when the basic reproduction number (${\mathbb {R}}_0$) is greater than one and disease-free equilibrium when the basic reproduction number is less than one respectively. This convergence is independent of the fractional orders and assumed initial conditions. The paper equally emphasized the outcome of altering the fractional orders, infection and recovery rates on the disease patterns. Similarly, we also remarked the importance of some key control measures to curtail the co-spread of double strains of dengue and COVID-19.

## Introduction

**Figure 1:**
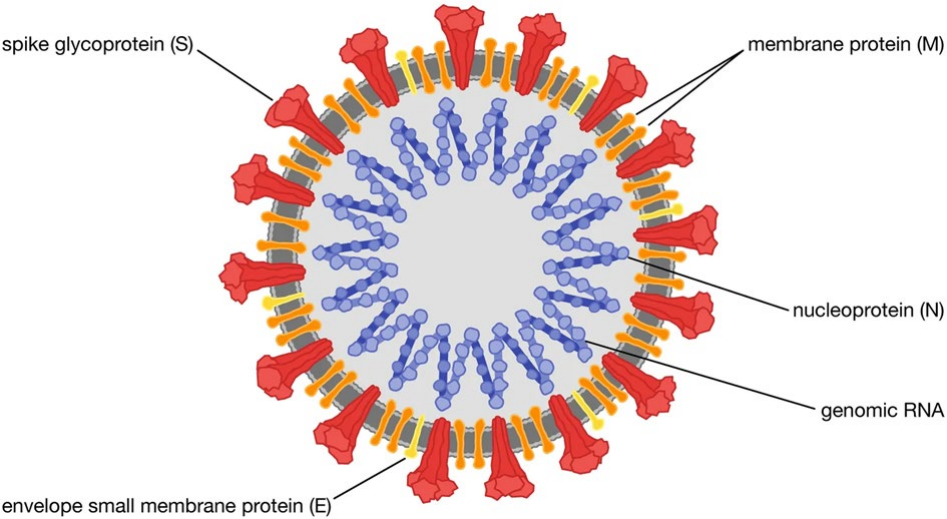
Circulation of COVID-19 infection.^7^.

The “Coronavirus Disease 2019” often referred to as COVID-19 is respiratory infection typically induced by the “severe acute respiratory coronavirus-2” SARS-COV-2^1^. Since the first incidence in 2019, approximately 514,943,711 individuals have been affected and over six million mortality recorded across the globe^2^. It is worthy to note that COVID-19 has become a prevalent disease. The transmission can be activated when an individual makes contact with a droplet from an infected person released through the mouth, nose or any other means^3^. Most often, the symptoms may include loss of smell, tiredness, cough, breathing difficulty, pains in the body and so on^4^. The evolution of the disease has led to emergence of recent variants such as omicron, delta, alpha etc invading at different rates, though the strains of omicron are suspected to be predominant^5^. Infected persons may exhibit mild, moderate or severe respiratory sickness depending on the body and immune system. While those with mild or moderate respiratory sickness may recover without special medical intervention, those with severe respiratory sickness will definitely require serious medical attention^3^. At the onset of the infection, efforts were made to reduce the spread of COVID-19 such as the use of face masks and self-isolation which culminated to lockdowns in many countries thereby crippling the economy. Further intervention was made to reduce the burden of this disease by developing a vaccine which has given hope to a possible end to the pandemic^6^ (Fig. 1).

**Figure 2:**
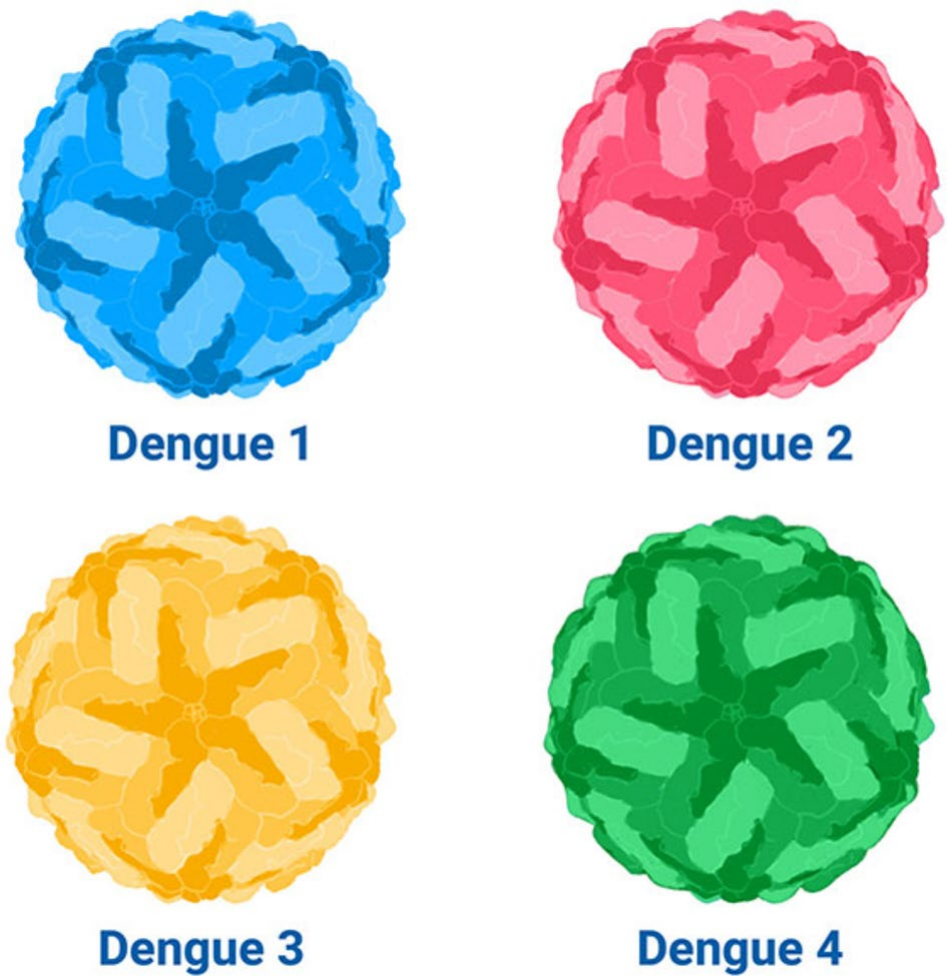
Circulation of Dengue strains.^13^.

On the other hand, dengue is an acute febrile (feverish) disease induced by dengue virus (DENV) and flavivirus. Dengue virus, which spreads through Aedes mosquitoes, thrives in tropical and sub-tropical regions. Dengue disease is now prevalent in over 100 countries with Asia accounting for about 70% of the disease concentration globally^8^. About 390 million individuals are predicted to be infected by dengue virus each year of which 96 million may show clinical manifestations^9^. Similarly, the WHO has reported 5.2 million cases of dengue in 2019^8^. The exact figures of dengue occurrence are mis-reported because most of the cases are mild and lacking symptoms^8^. Symptomatic cases manifest in the form of high fever, joint pain, rash, nausea etc^8,10^. Few of these symptomatic individuals may progress to a complicated fever known as “dengue hemorrhagic fever and “dengue shock syndrome”. Presently, there are four distinct strains of dengue virus and they include DENV-I, DENV-II, DENV-III and DENV-IV^1,11,12^. It is also pertinent to note that infection with one strain of dengue virus may not provide permanent or cross immunity over the other strains^11^ (Fig. 2).

**Figure 3:**
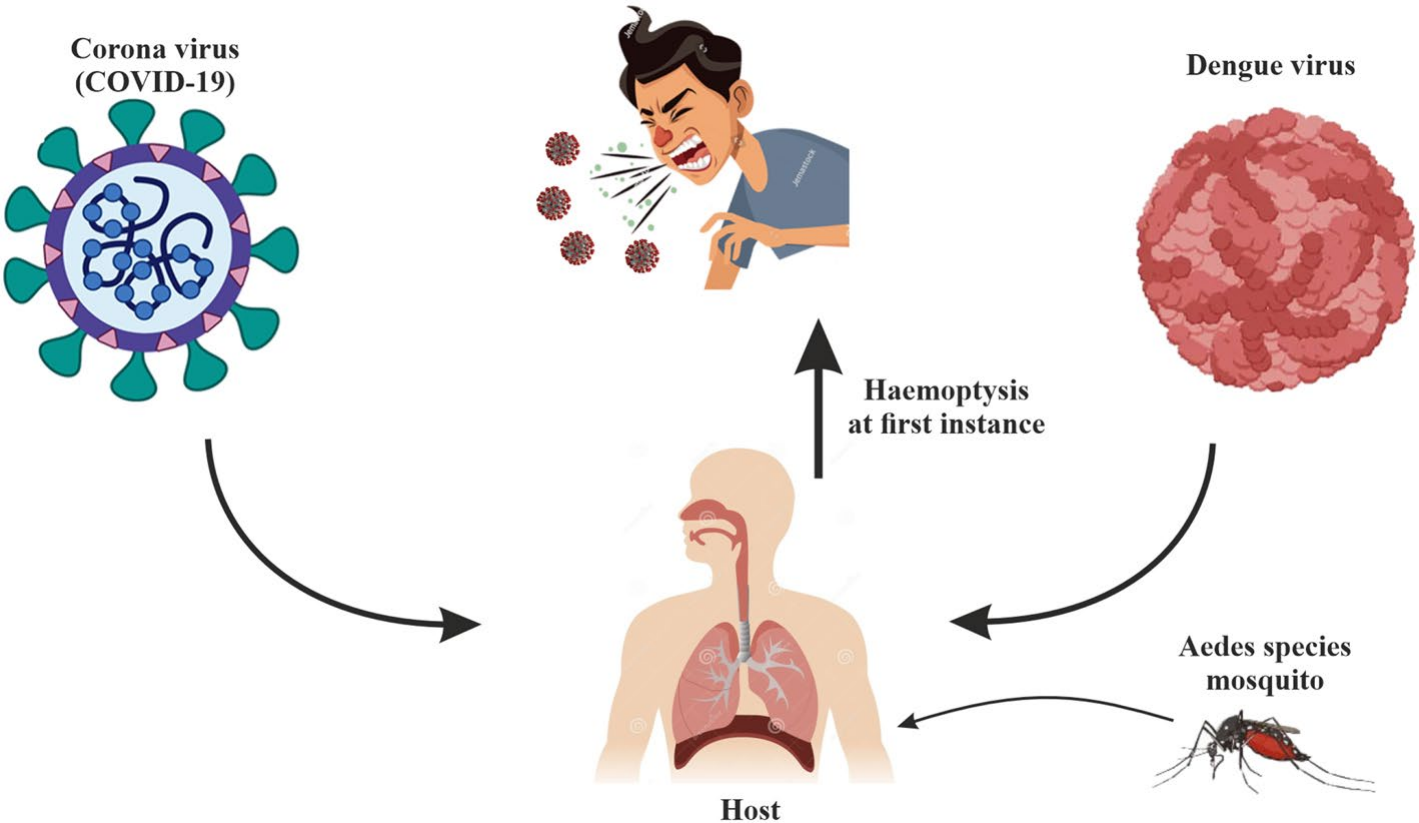
Co-circulation of COVID-19 and Dengue Virus.

Cases of co-infection of COVID-19 and dengue have been reported by the authors^14–17^. A co-infection of coronavirus and dengue virus invading a population can be life-threatening. This is due to the fact that co-morbidity cases in co-infection is more dangerous when compared to a sole viral infection^14^. Similarly, high mortality has been associated with individuals co-infected with dengue and coronavirus disease^15^. It has been reported that dengue-infected persons who are co-infected with coronavirus may suffer heightened sickness and hospitalization^2^. Despite the difference in pathophysiologies of the two diseases, the viruses can have impacts inside the body when compared thus, leading to indistinguishable clinical manifestations in a situation of co-infection which contributes to the complication^14^. As a result of corresponding symptoms between the two diseases, the chance of mis-diagnosis of the two infections is always high^16,18^. It is pertinent to note that increased glucose levels may manifest in individuals co-infected with dengue and coronavirus and this often leads to breeding of coronavirus^2^ (Fig. 3).

Mathematical modeling is a veritable tool for studying many biological systems such as the dynamics of diseases. Studies carried out in the past have greatly employed the classical integer order derivatives to gain useful insight in epidemiological models^6,19–22^. Some of these integer models have been used to understand the dynamics of Papillomavirus and tuberculosis^23,24^, COVID-19^25^, HIV and syphilis^26^, dengue disease^27^, zika virus^28^ and malaria infections^29^. Currently, authors in^30^ have examined the model for diabetes and tuberculosis in the direction of co-infection. The authors^1^ applied an integer model to analyze the triple infection of Zika, dengue and COVID-19. Also, Omame et al.^19^ studied the integer model of co-infection involving COVID-19 and dengue. They obtained an optimal strategy and cost effectiveness of controlling the co-infection. It is worthy to mention that, integer models were extensively used to understand the dynamics of COVID-19 at the early stage, in the year 2020^31–34^. More so, the enormous use of classical integer-order models to investigate the dynamics of dengue viral disease cannot be overemphasized^27,35–37^.

Despite the wide use of integer derivatives to model infectious diseases, they are limited due to the fact that they cannot easily capture memory effects. Meanwhile, memory effect relates to the fact that the future state of an operator due to the fact that they cannot on the current state and the past state of a given time-dependent system^38^. Hence, a proper understanding of the past dynamics of the disease could facilitate the control of the proliferation of the disease in future^39^. The inclusion of this memory property has motivated research with fractional differential equations. Fractional derivative is at the center stage of epidemiological modeling^2,4,38–45^. Currently, three fractional derivatives are commonly used to model infectious diseases. They include; Caputo derivative, Caputo-Fabrizio (CF) derivative and Atangana-Baleanu (AB) derivative. Many authors have used these derivatives in epidemiological modeling. According to authors in^46,47^, methods of Adomian decomposition, finite difference method (FDM), homotopy analysis method (HAM), spectral and homotopy perturbation have been proposed for solving fractional derivative models. Authors in^48^ proposed q-homotopy analysis Sumudu transform method (q-HASTM) for solving their fractional model. The authors in^4^ studied a model involving COVID-19 through a fractional derivative where the stability of the model was established in the context of Ulam-Hyers criteria. The model was numerically solved via the method of Adam-Bashforth Moulton. In^45^, the authors considered a COVID-19 model using a fractional-order derivative in Caputo-Fabrizio sense. They used the method of Laplace transform homotopy analysis to resolve the model. The authors in^49^ introduced a model using Atangana-Baleanu derivative for COVID-19 and tuberculosis and established the criteria for clearance of the two diseases and co-existence. Similarly, Omame et al.^39^ also considered Atangana-Baleanu fractional model involving double strains of COVID-19 and HIV as a co-infection. The model was resolved using the method of Laplace Adomian decomposition. Furthermore, Omame et al.^2^ proposed a model for triple infection of SARS-COV-2, HIV and dengue where they employed the method of Laplace Adomian decomposition to examine the model by using the three fractional derivatives of Caputo, CF and AB at specific fractional values. Likewise, authors in^50^ developed a composition of non-integer models to forecast new strains of COVID-19 and dengue in the direction of co-infection. Their three models were compared with Caputo, CF and AB derivatives respectively.

In spite of many studies done in modeling dengue and COVID-19 co-infection, there is need to holistically build a mathematical model that will investigate the co-infection of the disease involving two strains of dengue. Thus, this paper aims to formulate a novel non-integer mathematical model for double strains of dengue and COVID-19 co-circulation, study the existence and uniqueness of the solution of the given model and determine the parameters that impact the dynamics of the diseases. The non-standard finite difference(NSFD) scheme shall be adopted to analyze the solution of the designed model. Many authors have considered the non-standard finite difference scheme in the analysis of their models (see^40,51–56^). NSFD, is the most veritable tool in finding numerical solutions to fractional-order models^51^. This discretization scheme has been confirmed to possess interesting solution properties of an epidemic model, such as positivity, stability and other laws of conversation, giving it more preference over other existing methods of solution such as perturbation/decomposition schemes^40^. It is hoped that, this study will provide new paths for further research studies in epidemiological modelling.

### Preliminaries

#### Definition 1.1

^2,57^ The Caputo fractional derivative of order $\Im \in (0,1)$ can be defined as

1 $$\begin{aligned} ^{C}_{0}D_{t}^{\Im }z(t)=\frac{1}{\Gamma (n-\Im )}\int _0^t(t-s)^{n-\Im -1}z^{(n)}(s)ds, \end{aligned}$$

where *n* is a natural number satisfying $\Im \in (n-1, n)$ such that $\Gamma (\cdot )$ is a gamma function given by

$$\begin{aligned} \Gamma (\Im )=\int _0^{\infty }e^{-s}s^{\Im -1}ds. \end{aligned}$$

If $0<\Im <1$, then the above Caputo derivative becomes

$$\begin{aligned} ^{C}_{0}D_{t}^{\Im }z(t)=\frac{1}{\Gamma (1-\Im )}\int _0^t(t-s)^{-\Im }z^{\prime }(s)ds. \end{aligned}$$

#### Definition 1.2

^2,50^ The Riemann–Liouville fractional integral of a function *z* of order $\Im \in \mathbb {R^+}$ is defined as

2 $$\begin{aligned} I_t^{\Im }z(t)=\frac{1}{\Gamma (\Im )}\int _0^t(t-s)^{\Im -1}z(s)ds, \quad t>0. \end{aligned}$$

#### Definition 1.3

^2^ The Laplace transform, ${\mathcal {L}}$, of the Caputo derivative is defined as

3 $$\begin{aligned} {\mathcal {L}}\left\{ ^{C}_{0}D_{t}^{\Im }z(t)\right\} =s^{\Im }{\mathcal {L}}\left\{ z(s)\right\} -s^{\Im -1}z(0), \quad \Im \in (0, 1). \end{aligned}$$

#### Theorem 1.1

^58^ *Let* $\left( M, \Vert \cdot \Vert \right)$ *be a Banach space and* $T: M\rightarrow M$ *a contraction mapping with constant* $\kappa \in [0,1)$. *Then T has a unique fixed point in M, that is, there exists a unique point* $x^{\star } \in M$ *such that* $T(x^{\star })=x^{\star }$. *Furthermore, for arbitrary* $x_0\in M$, *the sequence* $\left\{ x_n\right\}$ *defined by* $x_{n+1}=T(x_n)$, $n=0, 1, 2,\cdot \cdot \cdot$ c*onverges strongly to* $x^{\star }.$

## Model formulation

Let ${N_{H}(t)}$ be the total number of persons. Then, ${N_{H}(t)}$ is partitioned into the following mutually exclusive compartments: Uninfected (susceptible) persons, $\left( {S_{H}(t)}\right)$, infected persons with dengue strain one, $\left( {I_{1D}(t)}\right)$, infected persons with dengue strain two, $\left( {I_{2D}(t)}\right)$, infected persons with Coronavirus, $\left( {I_{C}(t)}\right)$, co-infected persons with dengue strain one and coronavirus, $\left( {I_{1DC}(t)}\right)$, co-infected persons with dengue strain two and coronavirus, $\left( {I_{2DC}(t)}\right)$, persons who have recovered from both strains of dengue, $\left( {R_{D}(t)}\right)$ and persons who have recovered from coronavirus $\left( {R_{C}(t)}\right)$ such that;

$$\begin{aligned} {N_{H}(t)}={S_{H}(t)}+{I_{1D}(t)}+{I_{2D}(t)}+{I_{C}(t)}+{I_{1DC}(t)}+{I_{2DC}(t)}+{R_{D}(t)}+{R_{C}(t)}. \end{aligned}$$

Furthermore, Let ${N_{V}(t)}$ be the total number of vectors (mosquitoes). Then, ${N_{V}(t)}$ is partitioned into the following mutually exclusive compartments: Uninfected (susceptible) vector, $\left( {S_{V}(t)}\right)$, infected vectors with dengue strain one, $\left( {I_{1V}(t)}\right)$ and infected vectors with dengue strain two, $\left( {I_{2V}(t)}\right)$ such that;

$$\begin{aligned} {N_{V}(t)}={S_{V}(t)}+{I_{1V}(t)}+{I_{2V}(t)}. \end{aligned}$$

Uninfected persons, ${S_{H}}$ are recruited into the population at the rate ${\omega _{H}}$. The population is depleted as a result of infection with dengue strain one, dengue strain two and coronavirus respectively. This is due to effective contacts with an infected vector(with dengue strain one) at the rate ${\lambda _{1V}}$, infected vector (with dengue strain two) at the rate of ${\lambda _{2V}}$ and infected person with coronavirus at the rate of ${\lambda _{C}}$.

with,

${\lambda _{1V}}=\dfrac{{\beta _{1V}}{I_{1V}}}{{N_{H}}},~{\lambda _{2V}}=\dfrac{{\beta _{2V}}{I_{2V}}}{{N_{H}}},~{\lambda _{C}}=\dfrac{{\beta _{C}}\left( {I_{C}}+{I_{1DC}}+{I_{2DC}} \right) }{{N_{H}}}$, where ${\beta _{1V}}, ~{\beta _{2V}}, ~{\beta _{C}}$ denote effective contact rates between susceptible humans and infectious vector with dengue strain one, dengue strain two and infectious humans with coronavirus, respectively. Individuals who have recovered from dengue may lose their dengue-acquired immunity at the rate $\theta _D$ and becomes susceptible. Also, Individuals who have recovered from coronavirus may lose their coronavirus-acquired immunity at the rate $\theta _C$ and subsequently become susceptible. Natural mortality is assumed to be the same for all persons, at the rate ${\mu _{H}}$

Similarly, uninfected vectors acquire dengue strain one or strain two as a result of effective contact with infected persons with dengue strain one or two, respectively, at the rate

${\lambda _{1D}}=\dfrac{{\beta _{1D}}\left( {I_{1D}}+{I_{1DC}} \right) }{{N_{H}}}$ or ${\lambda _{2D}}=\dfrac{{\beta _{2D}}\left( {I_{2D}}+{I_{2DC}} \right) }{{N_{H}}}$. Vector removal is assumed at the rate ${\mu _{V}}$. Other parameters are detailed in Table 1.

The following assumptions are made in the formulation of the model:

i. Infected persons with coronavirus may contract dengue virus and vice versa.
ii. Co-infected persons may either transmit dengue virus or coronavirus but not the both diseases, simultaneously.
iii. Co-infected persons may either recover from dengue virus or coronavirus but not from both diseases,simultaneously.
iv. Individuals can only be infected with one strain of dengue but not both strains at the same time.
v. Transmission rate for an infected and co-infected person are assumed the same.

Thus, the model of Caputo fractional order, $\Im \in (0,1)$ is given by:

4 $$\begin{aligned} ^{C}_{0}D_{t}^{\Im }{S_{H}}&={\omega _{H}}-\left( {\lambda _{1V}}+{\lambda _{2V}}+{\lambda _{C}}\right) {S_{H}}-{\mu _{H}}{S_{H}}+{\theta _{D}}{R_{D}}+{\theta _{C}}{R_{C}}\\ ^{C}_{0}D_{t}^{\Im }{I_{1D}}&={\lambda _{1V}}\left( {S_{H}}+{R_{C}}\right) -\left( {\mu _{H}}+{\delta _{1D}}+{\uptau _{1D}}\right) {I_{1D}}-{\varepsilon _{C}}{\lambda _{C}}{I_{1D}}+{\uptau _{C}}{I_{1DC}}\\ ^{C}_{0}D_{t}^{\Im }{I_{2D}}&={\lambda _{2V}}\left( {S_{H}}+{R_{C}}\right) -\left( {\mu _{H}}+{\delta _{2D}}+{\uptau _{2D}}\right) {I_{2D}}-{\varepsilon _{C}}{\lambda _{C}}{I_{2D}}+{\uptau _{C}}{I_{2DC}}\\ ^{C}_{0}D_{t}^{\Im }{I_{C}}&={\lambda _{C}}\left( {S_{H}}+{R_{D}}\right) -\left( {\mu _{H}}+{\delta _{C}}+{\uptau _{C}}\right) {I_{C}}-\left( {\varepsilon _{1V}}{\lambda _{1V}}+{\varepsilon _{2V}}{\lambda _{2V}}\right) {I_{C}}+{\uptau _{1D}}{I_{1DC}}+{\uptau _{2D}}{I_{2DC}}\\ ^{C}_{0}D_{t}^{\Im }{I_{1DC}}&={\varepsilon _{C}}{\lambda _{C}}{I_{1D}}+{\varepsilon _{1V}}{\lambda _{1V}}{I_{C}}-\left( {\mu _{H}}+{\delta _{1D}}+{\delta _{C}}+{\uptau _{1D}}+{\uptau _{C}}\right) {I_{1DC}}\\ ^{C}_{0}D_{t}^{\Im }{I_{2DC}}&={\varepsilon _{C}}{\lambda _{C}}{I_{2D}}+{\varepsilon _{2V}}{\lambda _{2V}}{I_{C}}-\left( {\mu _{H}}+{\delta _{2D}}+{\delta _{C}}+{\uptau _{2D}}+{\uptau _{C}}\right) {I_{2DC}}\\ ^{C}_{0}D_{t}^{\Im }{R_{D}}&={\uptau _{1D}}{I_{1D}}+{\uptau _{2D}}{I_{2D}}-{\lambda _{C}}{R_{D}}-{\mu _{H}}{R_{D}}-{\theta _{D}}{R_{D}}\\ ^{C}_{0}D_{t}^{\Im }{R_{C}}&={\uptau _{C}}{I_{C}}-\left( {\lambda _{1V}}+{\lambda _{2V}}\right) {R_{C}}-{\mu _{H}}{R_{C}}-{\theta _{C}}{R_{C}}\\ ^{C}_{0}D_{t}^{\Im }{S_{V}}&={\omega _{V}}-\left( {\lambda _{1D}}+{\lambda _{2D}}\right) {S_{V}}-{\mu _{V}}{S_{V}}\\ ^{C}_{0}D_{t}^{\Im }{I_{1V}}&={\lambda _{1D}}{S_{V}}-{\mu _{V}}{I_{1V}}\\ ^{C}_{0}D_{t}^{\Im }{I_{2V}}&={\lambda _{2D}}{S_{V}}-{\mu _{V}}{I_{2V}} \end{aligned}$$

subject to the initial conditions-

$$\begin{aligned} {S_{H}}(0) & = S_{H0},\quad {I_{1D}}(0)={I_{1D0}},\quad {I_{2D}}(0)={I_{2D0}},\quad {I_{C}}(0)={I_{C0}},\quad {I_{1DC}}(0)={I_{1DC0}},\quad {I_{2DC}}(0)={I_{2DC0}},\\ \quad {R_{D}}(0) &= {R_{D0}},\quad {R_{C}}(0)={R_{C0}},\quad {S_{V}}(0)={S_{V0}},\quad {I_{1V}}(0)={I_{1V0}},\quad {I_{2V}}(0)={I_{2V0}}. \end{aligned}$$

**Table 1:** Description of parameters in the model (4).

Variables	Interpretation		
${S_{H}}$	Uninfected (susceptible) persons		
${I_{1D}}$	Infected persons with dengue strain one		
${I_{2D}}$	Infected persons with dengue strain two		
${I_{C}}$	Infected persons with coronavirus		
${I_{1DC}}$	Co-infected persons with dengue strain one and coronavirus		
${I_{2DC}}$	Co-infected persons with dengue strain two and coronavirus		
${R_{D}}$	Persons recovered from both dengue strains		
${R_{C}}$	Persons recovered from coronavirus		
${S_{V}}$	Uninfected (susceptible) vectors		
${I_{1V}}$	Infected vector with dengue strain one		
${I_{2V}}$	Infected vectors with dengue strain two		

Parameters	Interpretation	Value	References
${\omega _{H}}$	Constant recruitment rate for persons	$\frac{4,269,995}{74.90\times 365}$	^59^
${\omega _{V}}$	Constant recruitment rate for vectors	1500	^1^
${\mu _{H}}$	Natural death rate for persons	$\frac{1}{74.90\times 365}$per day	^59^
${\mu _{V}}$	vector removal rate	$\frac{1}{21}$ per day	^19^
${\theta _{D}}$	Acquired immunity loss to dengue	0.026	^19^
${\theta _{C}}$	Acquired immunity loss to coronavirus	0.00000043117	^19^
${\beta _{1D}}$	Transmission rate for human to vector spread of dengue strain one	0.60	^19^
${\beta _{2D}}$	Transmission rate for human to vector spread of dengue strain two	0.62	Assumed
${\beta _{C}}$	Coronavirus transmission rate	0.8368	Fitted
${\beta _{1V}}$	Transmission rate for vector to human spread of dengue strain one	0.0100	Fitted
${\beta _{2V}}$	Transmission rate for vector to human spread of dengue strain two	0.0747	Fitted
${\delta _{1D}}$	Dengue strain one-induced death rate	0.0012	Assumed
${\delta _{2D}}$	Dengue strain two-induced death rate	0.001	^19^
${\delta _{C}}$	coronavirus-induced death rate	0.0060	^19^
${\uptau _{1D}}$	Dengue strain one recovery rate	0.17	Assumed
${\uptau _{2D}}$	Dengue strain two recovery rate	0.15	^19^
${\uptau _{C}}$	coronavirus recovery rate	0.7031	Fitted
${\varepsilon _{1V}}$	Modification parameter accounting for susceptibility of coronavirus-infected persons to dengue strain one	1	Assumed
${\varepsilon _{2V}}$	Modification parameter accounting for susceptibility of coronavirus-infected persons to dengue strain two	1	Assumed
${\varepsilon _{C}}$	Modification parameter accounting for susceptibility of dengue-infected persons to coronavirus	1	Assumed

## Basic properties of the model

In order to examine the mathematical and biological well-posedness of the model (4), we shall establish the non-negativity of solution. Furthermore, we shall prove the existence and uniqueness of the solution to the model.

### Invariant regions

The dynamics of Caputo-Fractional model (4) is explored in the feasible region

$$\begin{aligned} \mho&=\mho _H\times \mho _V \subset {\mathbb {R}}^{11}_+\\ with\\ \mho _H&=\left\{ \left( {S_{H}}, {I_{1D}}, {I_{2D}}, {I_{C}}, {I_{1DC}}, {I_{2DC}}, {R_{D}}, {R_{C}} \right) \in {\mathbb {R}}^8_+:{S_{H}}+{I_{1D}}+{I_{2D}}+{I_{C}}+{I_{1DC}}+{I_{2DC}}+{R_{D}}+{R_{C}}\le \dfrac{{\omega _{H}}}{{\mu _{H}}} \right\} \\ and\\ \mho _V&=\left\{ \left( {S_{V}}, {I_{1V}}, {I_{2V}} \right) \in {\mathbb {R}}^3_+:{S_{V}}+{I_{1V}}+{I_{2V}}\le \dfrac{{\omega _{V}}}{{\mu _{V}}}\right\} \end{aligned}$$

#### Theorem 3.1

*The closed set* $\mho =\mho _H\times \mho _V \subset {\mathbb {R}}^{11}_+$ *is positively invariant subject to the nonnegative initial conditions with respect to model *(4).

#### Proof

Summing the component equations of the human population of model (4) yields

$$\begin{aligned} ^{C}_{0}D_{t}^{\Im }{N_{H}}={\omega _{H}}-{\mu _{H}}{N_{H}}-{\delta _{1D}}({I_{1D}}+{I_{1DC}})-{\delta _{2D}}({I_{2D}}+{I_{2DC}})-{\delta _{C}}({I_{C}}+{I_{1DC}}+{I_{2DC}}). \end{aligned}$$

The above equation can also be rewritten in the form of the inequality below:

$$\begin{aligned} ^{C}_{0}D_{t}^{\Im }{N_{H}}\le {\omega _{H}}-{\mu _{H}}{N_{H}} \end{aligned}$$

Applying Laplace and inverse Laplace transforms respectively to the inequality and simplifying, we obtain

5 $$\begin{aligned} {N_{H}}(t)\le {N_{H}}(0)E_{\Im }({-\mu _{H}}t^{\Im })+{\omega _{H}}t^{\Im }E_{\Im ,\Im +1}({-\mu _{H}}t^{\Im }), \end{aligned}$$

where, the “Mittag–Leffler function” is defined as

$$\begin{aligned} E_{\Im ,\beta }(x)=\sum _{j=0}^{\Im }\dfrac{x^j}{\Gamma (\Im j+\beta )}. \end{aligned}$$

Thus,

6 $$\begin{aligned} {N_{H}}(t)=\bigg ({N_{H}}(0)-\frac{{\omega _{H}}}{{\mu _{H}}} \bigg )E_{\Im }({-\mu _{H}}t^{\Im })+\frac{{\omega _{H}}}{{\mu _{H}}}. \end{aligned}$$

Considering that, $E_{\Im }({-\mu _{H}}t^{\Im }) \rightarrow 0$ as $t \rightarrow \infty$, we have that the total population of humans,

7 $$\begin{aligned} {N_{H}}(t)\le \frac{{\omega _{H}}}{{\mu _{H}}}~as~t\rightarrow \infty . \end{aligned}$$

Consequently, it can be shown that the population of vectors ${N_{V}}(t)\le \frac{{\omega _{V}}}{{\mu _{V}}}~as~t\rightarrow \infty$

Therefore, the closed set $\mho =\mho _H\times \mho _V \subset {\mathbb {R}}^{11}_+~is~ postively~ invariant$ $\square$

### Existence and uniqueness of solution

In this subsection, we shall prove the existence of a unique solution to model (4) from Banach fixed point theorem. Let $\psi (t)=({S_{H}},~{I_{1D}},~{I_{2D}},~{I_{C}},~{I_{1DC}},~{I_{2DC}},~{R_{D}},~{R_{C}},~{S_{V}},~{I_{1V}},~{I_{2V}})^T\in {\mathbb {R}}^{11}$ for $t\in [0,l]$ denote the state variables and $\zeta =(\zeta _1,\zeta _2,\zeta _3,\zeta _4,\zeta _5,\zeta _6,\zeta _7,\zeta _8,\zeta _9,\zeta _{10},\zeta _{11})^T$ represent a continuous vector given as follows;

$$\begin{aligned} \begin{pmatrix} \zeta _1\\ \zeta _2\\ \zeta _3\\ \zeta _4\\ \zeta _5\\ \zeta _6\\ \zeta _7\\ \zeta _8\\ \zeta _9\\ \zeta _{10}\\ \zeta _{11}\\ \end{pmatrix}= & {} \begin{pmatrix} {\omega _{H}}-\left( {\lambda _{1V}}+{\lambda _{2V}}+{\lambda _{C}}\right) {S_{H}}-{\mu _{H}}{S_{H}}+{\theta _{D}}{R_{D}}+{\theta _{C}}{R_{C}}\\ {\lambda _{1V}}\left( {S_{H}}+{R_{C}}\right) -\left( {\mu _{H}}+{\delta _{1D}}+{\uptau _{1D}}\right) {I_{1D}}-{\varepsilon _{C}}{\lambda _{C}}{I_{1D}}+{\uptau _{C}}{I_{1DC}}\\ {\lambda _{2V}}\left( {S_{H}}+{R_{C}}\right) -\left( {\mu _{H}}+{\delta _{2D}}+{\uptau _{2D}}\right) {I_{2D}}-{\varepsilon _{C}}{\lambda _{C}}{I_{2D}}+{\uptau _{C}}{I_{2DC}}\\ {\lambda _{C}}\left( {S_{H}}+{R_{D}}\right) -\left( {\mu _{H}}+{\delta _{C}}+{\uptau _{C}}\right) {I_{C}}-\left( {\varepsilon _{1V}}{\lambda _{1V}}+{\varepsilon _{2V}}{\lambda _{2V}}\right) {I_{C}}+{\uptau _{1D}}{I_{1DC}}+{\uptau _{2D}}{I_{2DC}}\\ {\varepsilon _{C}}{\lambda _{C}}{I_{1D}}+{\varepsilon _{1V}}{\lambda _{1V}}{I_{C}}-\left( {\mu _{H}}+{\delta _{1D}}+{\delta _{C}}+{\uptau _{1D}}+{\uptau _{C}}\right) {I_{1DC}}\\ {\varepsilon _{C}}{\lambda _{C}}{I_{2D}}+{\varepsilon _{2V}}{\lambda _{2V}}{I_{C}}-\left( {\mu _{H}}+{\delta _{2D}}+{\delta _{C}}+{\uptau _{2D}}+{\uptau _{C}}\right) {I_{2DC}}\\ {\uptau _{1D}}{I_{1D}}+{\uptau _{2D}}{I_{2D}}-{\lambda _{C}}{R_{D}}-{\mu _{H}}{R_{D}}-{\theta _{D}}{R_{D}}\\ {\uptau _{C}}{I_{C}}-\left( {\lambda _{1V}}+{\lambda _{2V}}\right) {R_{C}}-{\mu _{H}}{R_{C}}-{\theta _{C}}{R_{C}}\\ {\omega _{V}}-\left( {\lambda _{1D}}+{\lambda _{2D}}\right) {S_{V}}-{\mu _{V}}{S_{V}}\\ {\lambda _{1D}}{S_{V}}-{\mu _{V}}{I_{1V}}\\ {\lambda _{2D}}{S_{V}}-{\mu _{V}}{I_{2V}}\\ \end{pmatrix} \end{aligned}$$

Thus, model (2.1) can be re-written as

8 $$\begin{aligned} {\left\{ \begin{array}{ll} ^{C}_{0}D_{t}^{\Im }\varphi (t)=\zeta (t,\varphi (t))\\ \varphi (0)~~~~=\varphi _0 \end{array}\right. } \end{aligned}$$

Furthermore, $\zeta =[0,l]\times {\mathbb {R}}^{11}\rightarrow {\mathbb {R}}^{11}$ is said to be Lipschitz with respect to the second argument, if the following inequality holds

9 $$\begin{aligned} \Vert \zeta (t,\varphi _1)-\zeta (t,\varphi _2)\Vert \le K\Vert \varphi _1-\varphi _2\Vert ~\forall ~t\in [0,l], \varphi _1, \varphi _2 \in {\mathbb {R}}^{11} \end{aligned}$$

where $K>0$ is the Lipschitz constant

#### Theorem 3.2

*Suppose Eq.* (9) *is satisfied and* ${\mathcal {M}}K<1$, *with* ${\mathcal {M}}=\dfrac{l^{\Im }}{\Gamma (\Im +1)}$, *then, there exists a unique solution to the initial value problem* (8) *on* $C\big ( [0,l],{\mathbb {R}}^{11}\big )$

#### Proof

Applying the Caputo fractional integral on both sides of (8) , we obtain

$$\varphi (t)=\varphi _0+\frac{1}{\Gamma (\Im )}\int _0^t(t-s)^{\Im -1}\zeta (s,\varphi (s))ds$$.

We define an operator $\varrho :C\big (g,{\mathbb {R}}^{11}\big )\rightarrow C\big (g,{\mathbb {R}}^{11}\big )$ by:

$\varrho [\varphi ](t)=U(t).~\varphi , U\in C\big (g,{\mathbb {R}}^{11}\big )$ where $g=[0,l]$ and with

$U(t)=\varphi _0+\frac{1}{\Gamma (\Im )}\int _0^t(t-s)^{\Im -1}\zeta (s,\varphi (s))ds$ endowed with the supremum norm

$$\Vert U\Vert =\sup _{t\in g}\Vert U(t)\Vert ,~\forall ~U\in C(g, {\mathbb {R}}^{11})$$

Thus, $C(g, {\mathbb {R}}^{11})$ endowed with $\Vert .\Vert$ is a Banach space. It suffices to show that the operator

$\varrho :C\big (g,{\mathbb {R}}^{11}\big )\rightarrow C\big (g,{\mathbb {R}}^{11}\big )$ is a contraction mapping.

Now,

$$\begin{aligned} \Vert \varrho [U](t)-\varrho [V](t)\Vert{} & {} = \bigg \Vert \varphi _0+\frac{1}{\Gamma (\Im )}\int _0^t(t-s)^{\Im -1}\zeta (s,U(s))ds-\varphi _0\\{} & {} \quad +\frac{1}{\Gamma (\Im )}\int _0^t(t-s)^{\Im -1}\zeta (s,V(s))ds \bigg \Vert \\{} & {} \le \parallel \frac{1}{\Gamma (\Im )}\int _0^t(t-s)^{\Im -1}[\zeta (s,U(s))-\zeta (s,V(s))]ds\parallel \\{} & {} \le \frac{1}{\Gamma (\Im )}\parallel \int _0^t(t-s)^{\Im -1}[\zeta (s,U(s))-\zeta (s,V(s))]ds\parallel \end{aligned}$$

since the operator $\zeta$ satisfies Lipschitz condition, we have that

$$\begin{aligned} \frac{1}{\Gamma (\Im )} \parallel \int _0^t(t-s)^{\Im -1}[\zeta (s,U(s))-\zeta (s,V(s))]ds\parallel \le \frac{K}{\Gamma (\Im )}\int _0^t(t-s)^{\Im -1} \parallel U(s)-V(s)ds\parallel \end{aligned}$$

By taking the supremum over $t\in g=[0,l]$ we see that

$$\begin{aligned} \frac{K}{\Gamma (\Im )}\int _0^t(t-s)^{\Im -1} \parallel U(s)-V(s)ds\parallel\le & {} \frac{K}{\Gamma (\Im )}sup_{t\in g}\int _0^t(t-s)^{\Im -1} \parallel U(s)-V(s)ds\parallel \\\le & {} {\mathcal {M}}K\parallel U-V\parallel \end{aligned}$$

If $${\mathcal {M}}K<1$$ then, $$\parallel \varrho [U]-\varrho [V]\parallel \le {\mathcal {M}}K\parallel U-V\parallel$$

Thus, $\varrho$ is a contraction and by the Banach contraction mapping principle, $\varrho$ has a unique fixed point which is a solution to the initial value problem (8) and thus the solution to the model (4).

## Stability analysis of the model

### The basic reproduction number of the model

By setting the right-hand sides of model (4) to zero we obtain the disease-free equilibrium (DFE) as

$$\begin{aligned} {\mathcal {D}}_0=\big ({S_{H}^{\star }},0,0,0,0,0,0,0,{S_{V}^{\star }},0,0\big )=\bigg (\frac{{\omega _{H}}}{{\mu _{H}}},0,0,0,0,0,0,0,\frac{{\omega _{V}}}{{\mu _{V}}},0,0\bigg ). \end{aligned}$$

The stability of the DFE is analysed by applying the next generation matrix on model (4). The respective transfer matrices are given below;

$$\begin{aligned} F= \begin{pmatrix} 0&{}0&{}0&{}0&{}0&{}{\beta _{1V}}&{}0\\ 0&{}0&{}0&{}0&{}0&{}0&{}{\beta _{2V}}\\ 0&{}0&{}{\beta _{C}}&{}{\beta _{C}}&{}{\beta _{C}}&{}0&{}0\\ 0&{}0&{}0&{}0&{}0&{}0&{}0\\ 0&{}0&{}0&{}0&{}0&{}0&{}0\\ \frac{{\beta _{1D}}{S_{V}^{\star }}}{{S_{H}^{\star }}}&{}0&{}0&{}\frac{{\beta _{1D}}{S_{V}^{\star }}}{{S_{H}^{\star }}}&{}0&{}0&{}0\\ 0&{}\frac{{\beta _{2D}}{S_{V}^{\star }}}{{S_{H}^{\star }}}&{}0&{}0&{}\frac{{\beta _{2D}}{S_{V}^{\star }}}{{S_{H}^{\star }}}&{}0&{}0 \end{pmatrix}, \quad V= \begin{pmatrix} q_1&{}0&{}0&{}-{\tau _{C}}&{}0&{}0&{}0\\ 0&{}q_2&{}0&{}0&{}-{\tau _{C}}&{}0&{}0\\ 0&{}0&{}q_3&{}-{\tau _{1D}}&{}-{\tau _{2D}}&{}0&{}0\\ 0&{}0&{}0&{}q_4&{}0&{}0&{}0\\ 0&{}0&{}0&{}0&{}q_5&{}0&{}0\\ 0&{}0&{}0&{}0&{}0&{}{\mu _{V}}&{}0\\ 0&{}0&{}0&{}0&{}0&{}0&{}{\mu _{V}} \end{pmatrix} \end{aligned}$$

with,

$$q_1={\delta _{1D}}+{\mu _{H}}+{\tau _{1D}},~q_2= {\delta _{2D}}+{\mu _{H}}+{\tau _{2D}}, ~q_3={\delta _{C}}+{\mu _{H}}+{\tau _{C}}, ~q_4={\delta _{1D}}+{\mu _{H}}+{\tau _{1D}}+{\delta _{C}}+{\tau _{C}},$$

$$~~~~~~~q_5={\delta _{2D}}+{\mu _{H}}+{\tau _{2D}}+{\delta _{C}}+{\tau _{C}}$$

Hence, the basic reproduction number of model (4) is given as follows,

${\mathcal {R}}_0=\rho (FV^{-1})=max\lbrace {\mathcal {R}}_{01D}, {\mathcal {R}}_{02D}, {\mathcal {R}}_{0C}\rbrace$ where ${\mathcal {R}}_{01D}$, ${\mathcal {R}}_{02D}$ and ${\mathcal {R}}_{0C}$ are the respective associated reproduction numbers for Dengue strain one, Dengue strain two and COVID-19 with

$${\mathcal {R}}_{01D}=\dfrac{1}{{\mu _{V}}}\sqrt{\dfrac{{\beta _{1D}}{\beta _{1V}}{\mu _{H}}{\omega _{V}}}{{\omega _{H}}({\delta _{1D}}+{\mu _{H}}+{\tau _{1D}})}}$$,    $${\mathcal {R}}_{02D}=\dfrac{1}{{\mu _{V}}}\sqrt{\dfrac{{\beta _{2D}}{\beta _{2V}}{\mu _{H}}{\omega _{V}}}{{\omega _{H}}({\delta _{2D}}+{\mu _{H}}+{\tau _{2D}})}}$$    and    $${\mathcal {R}}_{0C}=\dfrac{{\beta _{C}}}{{\delta _{C}}+{\mu _{H}}+{\tau _{C}}}$$

The above basic reproduction numbers, can be interpreted epidemiologically, as the average number of secondary infections caused by an infected individual with COVID-19 or dengue (strain one or strain two) in entirely susceptible population^60^.

### Local stability of the disease-free equilibrium (DFE) of the model

#### Theorem 4.1

*The model’s DFE* (${\mathcal {D}}_0$) *is locally asymptotically stable whenever* ${\mathcal {R}}_0 <1$ *and unstable when* ${\mathcal {R}}_0>1$

#### Proof

Analysis of the system around the infection-free equilibrium is done with the help of the Jacobian matrix of system (8) evaluated at the DFE, which is given by:

$$\begin{aligned} J({\mathcal {D}}_0)= \left( \begin{array}{lllllllllll} {}-{\mu _{H}}&{}0&{}0&{}-{\beta _{C}}&{}-{\beta _{C}}&{}-{\beta _{C}}&{}{\theta _{D}}&{}{\theta _{C}}&{}0&{}-{\beta _{1V}}&{}-{\beta _{2V}}\\ 0&{}-q_1&{}0&{}0&{}{\tau _{C}}&{}0&{}0&{}0&{}0&{}{\beta _{1V}}&{}0\\ 0&{}0&{}-q_2&{}0&{}0&{}{\tau _{C}}&{}0&{}0&{}0&{}0&{}{\beta _{2V}}\\ 0&{}0&{}0&{}-q_3+{\beta _{C}}&{}{\tau _{1D}}+{\beta _{C}}&{}{\tau _{2D}}+{\beta _{C}}&{}0&{}0&{}0&{}0&{}0\\ 0&{}0&{}0&{}0&{}-q_4&{}0&{}0&{}0&{}0&{}0&{}0\\ 0&{}0&{}0&{}0&{}0&{}-q_5&{}0&{}0&{}0&{}0&{}0\\ 0&{}{\tau _{1D}}&{}{\tau _{2D}}&{}0&{}0&{}0&{}-{\mu _{H}}-{\theta _{D}}&{}0&{}0&{}0&{}0\\ 0&{}0&{}0&{}{\tau _{C}}&{}0&{}0&{}0&{}-{\mu _{H}}-{\theta _{C}}&{}0&{}0&{}0\\ 0&{}-\frac{{\beta _{1D}}{S_{V}^\star }}{{S_{H}^\star }}&{}-\frac{{\beta _{2D}}{S_{V}^\star }}{{S_{H}^\star }}&{}0&{}-\frac{{\beta _{1D}}{S_{V}^\star }}{{S_{H}^\star }}&{}-\frac{{\beta _{2D}}{S_{V}^\star }}{{S_{H}^\star }}&{}0&{}0&{}-{\mu _{V}}&{}0&{}0\\ 0&{}\frac{{\beta _{1D}}{S_{V}^\star }}{{S_{H}^\star }}&{}0&{}0&{}\frac{{\beta _{1D}}{S_{V}^\star }}{{S_{H}^\star }}&{}0&{}0&{}0&{}0&{}-{\mu _{V}}&{}0\\ 0&{}0&{}\frac{{\beta _{2D}}{S_{V}^\star }}{{S_{H}^\star }}&{}0&{}0&{}\frac{{\beta _{2D}}{S_{V}^\star }}{{S_{H}^\star }}&{}0&{}0&{}0&{}0&{}-{\mu _{V}} \end{array}\right) \end{aligned}$$

with the eigenvalues given as follows;

$${\Lambda _{1}}=-{\mu _{H}},~~{\Lambda _{2}}=-{\mu _{V}},~~{\Lambda _{3}}=-({\delta _{1D}}+{\mu _{H}}+{\tau _{1D}}+{\delta _{C}}+{\tau _{C}}),~~{\Lambda _{4}}=-({\delta _{2D}}+{\mu _{H}}+{\tau _{2D}}+{\delta _{C}}+{\tau _{C}}),$$

$~{\Lambda _{5}}=-({\mu _{H}}+{\theta _{C}}),{\Lambda _{6}}=-({\mu _{H}}+{\theta _{D}})$. Clearly, $\Lambda _i<0$, $i=1, 2, 3, \cdot \cdot \cdot ,6$. The remaining eigenvalues can be found from the following characteristics polynomial equations

$$\Lambda +{q_{3}}\left( 1-{\mathcal {R}}_{0C}\right) =0$$

$$\Lambda ^2+{\mu _{V}}{q_{1}}\Lambda +{\mu _{V}}{q_{1}}\left( 1-{\mathcal {R}}_{01D}^2\right) =0$$

$$\Lambda ^2+{\mu _{V}}{q_{2}}\Lambda +{\mu _{V}}{q_{2}}\left( 1-{\mathcal {R}}_{02D}^2\right) =0$$

From Routh–Hurwitz criterion, the above three equations will have roots of negative real parts provided that the associated reproduction numbers ${\mathcal {R}}_{0C},~{\mathcal {R}}_{01D}$ and ${\mathcal {R}}_{02D}$ are less than one. Thus, the DFE, ${\mathcal {D}}_0$ is locally asymptotically stable whenever the reproduction number,

${\mathcal {R}}_0=max\left\{ {\mathcal {R}}_{0C},~{\mathcal {R}}_{01D}~{\mathcal {R}}_{02D}\right\} <1$ and otherwise if ${\mathcal {R}}_0>1$. Also, $\vert arg(\Lambda _i)\vert >\frac{\Im \pi }{2},$ where $\Im \in (0,1)$ and $Im(\Lambda _i)=0,$ for $i=0,1,2,\cdot \cdot \cdot ,11$

### Global asymptotic stability of the disease-free equilibrium (DFE) of the model (a special case)

We shall establish the global asymptotic stability of the DFE of the model for a special case. That is, in the absence of co-infection, re-infection and acquired immunity loss to COVID-19 and dengue $\left( I_{1DC}=I_{2DC}=\varepsilon _{1V}=\varepsilon _{2V}=\varepsilon _C={\theta _{D}}={\theta _{C}}=0\right)$. To achieve the global stability, we shall apply the direct Lyapunov method^61^.

#### Theorem 4.2

*Suppose there is no co-infection, re-infection and acquired immunity loss to COVID-19 and dengue in the model then, the DFE* (${\mathcal {D}}_0$) *of the model is global asymptotically stable (GAS) in* $\mho$ *whenever* ${\mathcal {R}}_0< 1.$

#### Proof

Consider a modified version of the model when both diseases are present in the population but, no co-infection of the two diseases, re-infection and acquired immunity loss to COVID-19 and dengue.

10 $$\begin{aligned} ^{C}_{0}D_{t}^{\Im }{S_{H}}&={\omega _{H}}-\left( \dfrac{{\beta _{1V}}{I_{1V}}}{{N_{H}}}+\dfrac{{\beta _{2V}}{I_{2V}}}{{N_{H}}}+\dfrac{{\beta _{C}}{I_{C}}}{{N_{H}}}\right) {S_{H}}-{\mu _{H}}{S_{H}}\\ ^{C}_{0}D_{t}^{\Im }{I_{1D}}&=\dfrac{{\beta _{1V}}{I_{1V}}}{{N_{H}}}{S_{H}}-\left( {\mu _{H}}+{\delta _{1D}}+{\uptau _{1D}}\right) {I_{1D}}\\ ^{C}_{0}D_{t}^{\Im }{I_{2D}}&=\dfrac{{\beta _{2V}}{I_{2V}}}{{N_{H}}}{S_{H}}-\left( {\mu _{H}}+{\delta _{2D}}+{\uptau _{2D}}\right) {I_{2D}}\\ ^{C}_{0}D_{t}^{\Im }{I_{C}}&=\dfrac{{\beta _{C}}{I_{C}} }{{N_{H}}}{S_{H}}-\left( {\mu _{H}}+{\delta _{C}}+{\uptau _{C}}\right) {I_{C}}\\ ^{C}_{0}D_{t}^{\Im }{R_{D}}&={\uptau _{1D}}{I_{1D}}+{\uptau _{2D}}{I_{2D}}-{\mu _{H}}{R_{D}}\\ ^{C}_{0}D_{t}^{\Im }{R_{C}}&={\uptau _{C}}{I_{C}}-{\mu _{H}}{R_{C}}\\ ^{C}_{0}D_{t}^{\Im }{S_{V}}&={\omega _{V}}-\left( \dfrac{{\beta _{1D}}{I_{1D}}}{{N_{H}}}+\dfrac{{\beta _{2D}}{I_{2D}}}{{N_{H}}}\right) {S_{V}}-{\mu _{V}}{S_{V}}\\ ^{C}_{0}D_{t}^{\Im }{I_{1V}}&=\dfrac{{\beta _{1D}}{I_{1D}}}{{N_{H}}}{S_{V}}-{\mu _{V}}{I_{1V}}\\ ^{C}_{0}D_{t}^{\Im }{I_{2V}}&=\dfrac{{\beta _{2D}}{I_{2D}}}{{N_{H}}}{S_{V}}-{\mu _{V}}{I_{2V}} \end{aligned}$$

Now, we consider the following Lyapunov function, from the approach^1,34,62,63^.

$${\mathbb {L}}_1=\dfrac{{\beta _{1D}}S^{\star }_V}{q_1{\mu _{V}}S_H^{\star }}{I_{1D}}+\dfrac{{\beta _{2D}}S^{\star }_V}{q_2{\mu _{V}}S_H^{\star }}{I_{2D}}+\dfrac{1}{q_3}{I_{C}}+\dfrac{{\mathcal {R}}_{01D}}{{\mu _{V}}}{I_{1V}}+\dfrac{{\mathcal {R}}_{02D}}{{\mu _{V}}}{I_{2V}}$$

with Caputo fractional derivative,

$$\begin{aligned} ^{C}_{0}D_{t}^{\Im }{\mathbb {L}}_1= & {} \dfrac{{\beta _{1D}}S^{\star }_V}{q_1{\mu _{V}}{S_{H}^{*}}}\left[ \dfrac{{\beta _{1V}}{I_{1V}}}{{N_{H}}}{S_{H}}-q_1{I_{1D}}\right] +\dfrac{{\beta _{2D}}S^{\star }_V}{q_2{\mu _{V}}{S_{H}^{**}}}\left[ \dfrac{{\beta _{2V}}{I_{2V}}}{{N_{H}}}{S_{H}}-q_2{I_{2D}}\right] +\dfrac{1}{q_3}\left[ \dfrac{{\beta _{C}}{I_{C}}}{{N_{H}}}{S_{H}}-q_3{I_{C}}\right] \\+ & {} \dfrac{{\mathcal {R}}_{01D}}{{\mu _{V}}}\left[ \dfrac{{\beta _{1D}}{I_{1D}}}{{N_{H}}}{S_{V}}-{\mu _{V}}{I_{1V}}\right] +\dfrac{{\mathcal {R}}_{02D}}{{\mu _{V}}}\left[ \dfrac{{\beta _{2D}}{I_{2D}}}{{N_{H}}}{S_{V}}-{\mu _{V}}{I_{2V}}\right] .\end{aligned}$$

Recalling that $${S_{H}}\le S^{\star }_H={N_{H}^{*}},\quad {S_{V}}\le S^{\star }_V,\quad {S_{H}}+{I_{1D}}+{I_{2D}}+{I_{C}}+{I_{1DC}}+{I_{2DC}}+{R_{D}}+{R_{C}}\le \dfrac{{\omega _{H}}}{{\mu _{H}}},\quad {S_{V}}+{I_{1V}}+{I_{2V}}\le \dfrac{{\omega _{V}}}{{\mu _{V}}}.$$

Thus,

$$\begin{aligned} ^{C}_{0}D_{t}^{\Im }{\mathbb {L}}_1\le & {} \dfrac{{\beta _{1D}}{S_{V}^{\star }}}{q_1{\mu _{V}}S_H^{\star }}\left( {\beta _{1V}}{I_{1V}}-q_1{I_{1D}}\right) +\dfrac{{\beta _{2D}}S^{\star }_V}{q_2{\mu _{V}}S_H^{\star }}\left( {\beta _{2V}}{I_{2V}}-q_2{I_{2D}}\right) +\dfrac{1}{q_3}\left( {\beta _{C}}{I_{C}}-q_3{I_{C}}\right) +\dfrac{{\mathcal {R}}_{01D}}{{\mu _{V}}}\left( \dfrac{{\beta _{1D}}{I_{1D}}{S_{V}^{\star }}}{{S_{H}^{*}}}-{\mu _{V}}{I_{1V}}\right) \\+ & {} \dfrac{{R_{02D}}}{{\mu _{V}}}\left( \dfrac{{\beta _{2D}}{I_{2D}}S_V^{\star }}{{S_{H}^{*}}}-{\mu _{V}}{I_{2V}}\right) \\= & {} \dfrac{{\beta _{1D}}S^{\star }_V}{{\mu _{V}}S_H^{\star }}\left( {\mathcal {R}}_{01D}-1\right) {I_{1D}}+\left[ \dfrac{{\beta _{1D}}{\beta _{1V}}{\mu _{H}}{\omega _{V}}}{\mu _V^2\omega _H\left( {\delta _{1D}}+{\mu _{H}}+{\uptau _{1D}}\right) }-{\mathcal {R}}_{01D}\right] {I_{1V}}+\dfrac{{\beta _{2D}}S^{\star }_V}{{\mu _{V}}S_H^{\star }}\left( {\mathcal {R}}_{02D}-1\right) {I_{2D}}\\+ & {} \left[ \dfrac{{\beta _{2D}}{\beta _{2V}}{\mu _{H}}{\omega _{V}}}{\mu _V^2\omega _H\left( {\delta _{2D}}+{\mu _{H}}+{\uptau _{2D}}\right) }-{R_{02D}}\right] {I_{2V}}+\left( {\mathcal {R}}_{0C}-1\right) {I_{C}}\\= & {} \dfrac{{\beta _{1D}}S^{\star }_V}{{\mu _{V}}S_H^{\star }}\left( {\mathcal {R}}_{01D}-1\right) {I_{1D}}+{\mathcal {R}}_{01D}\left( {\mathcal {R}}_{01D}-1\right) {I_{1V}}+\dfrac{{\beta _{2D}}S^{\star }_V}{{\mu _{V}}S_H^{\star }}\left( {\mathcal {R}}_{02D}-1\right) {I_{2D}}+{\mathcal {R}}_{02D}\left( {\mathcal {R}}_{02D}-1\right) {I_{2V}}+\left( {\mathcal {R}}_{0C}-1\right) {I_{C}} \end{aligned}$$

Thus, $^{C}_{0}D_{t}^{\Im }{\mathbb {L}}_1\le 0$ whenever ${\mathcal {R}}_0=max\lbrace {\mathcal {R}}_{01D}, {\mathcal {R}}_{02D}, {\mathcal {R}}_{0C}\rbrace <1$ and $^{C}_{0}D_{t}^{\Im }{\mathbb {L}}_1=0$ is equivalent to ${I_{1D}}={I_{2D}}={I_{C}}={I_{1DC}}={I_{2DC}}={I_{1V}}={I_{2V}}=0.$ Furthermore, the variables and parameters of the model are non-negative. Hence, ${\mathbb {L}}_1$ is an appropriate Lyapunov function on $\mho$. Thus, by Lassel’s invariance principle^61^, $\left( {I_{1D}}, {I_{2D}}, {I_{C}}, {I_{1DC}}, {I_{2DC}}, {I_{1V}}, {I_{2V}} \right) \rightarrow \left( 0, 0, 0, 0, 0, 0, 0\right)$ as $t\rightarrow \infty .$ By substituting ${I_{1D}}={I_{2D}}={I_{C}}={I_{1DC}}={I_{2DC}}={I_{1V}}={I_{2V}}=0$ in model (4), we obtain that ${R_{D}}\rightarrow 0, {R_{C}}\rightarrow 0, {S_{H}}\rightarrow S_H^{\star }, {S_{V}}\rightarrow S_V^{\star }$ as $t\rightarrow \infty .$ Therefore, every solution to model (4) having initial conditions in $\mho ,$ and with ${\varepsilon _{1V}}={\varepsilon _{2V}}={\varepsilon _{C}}={\theta _{D}}={\theta _{C}}=0$ approaches the DFE as $t\rightarrow \infty$, provided that ${\mathcal {R}}_0< 1.$ Epidemiologically, in the absence of the co-infection, re-infection and acquired immunity loss to COVID-19 and dengue, both infections can be eradicated when ${\mathcal {R}}_0< 1,$ irrespective of the initial quantities of the sub-populations.

### Global asymptotic stability of the disease-present equilibrium (DPE) of the model (a special case)

#### Theorem 4.3

*Suppose there is no co-infection, re-infection and acquired immunity loss to COVID-19 and dengue in the model* (4) *then, the DPE* (${\mathcal {D}}_0$) *of the model is global asymptotically stable (GAS) in* $\mho$ *whenever* ${\mathcal {R}}_0> 1.$

#### Proof

Consider a special case when there is no co-infection, re-infection with the same or different infection in the model and acquired immunity loss to COVID-19 and dengue. That is, $I_{1DC}=I_{2DC}\varepsilon _{1V}=\varepsilon _{2V}=\varepsilon _C={\theta _{D}}={\theta _{C}}=0$. Following the approach^1,34,63,64^, we consider the potential Lyapunov function constructed below.

$$\begin{aligned} {\mathbb {L}}_2&={S_{H}}-{S_{H}^{**}}-{S_{H}^{**}}\ln \left( \dfrac{{S_{H}}}{{S_{H}^{**}}}\right) +{I_{1D}}-{I_{1D}^{**}}-{I_{1D}^{**}}\ln \left( \dfrac{{I_{1D}}}{{I_{1D}^{**}}}\right) +{I_{2D}}-{I_{2D}^{**}}-{I_{2D}^{**}}\ln \left( \dfrac{{I_{2D}}}{{I_{2D}^{**}}}\right) +{I_{C}}-{I_{C}^{**}}-{I_{C}^{**}}\ln \left( \dfrac{{I_{C}}}{{I_{C}^{**}}}\right) \\&+{S_{V}}-{S_{V}^{**}}-{S_{V}^{**}}\ln \left( \dfrac{{S_{V}}}{{S_{V}^{**}}}\right) +{I_{1V}}-{I_{1V}^{**}}-{I_{1V}^{**}}\ln \left( \dfrac{{I_{1V}}}{{I_{1V}^{**}}}\right) +{I_{2V}}-{I_{2V}^{**}}-{I_{2V}^{**}}\ln \left( \dfrac{{I_{2V}}}{{I_{2V}^{**}}}\right) \end{aligned}$$

with Lyapunov Caputo derivative,

11 $$\begin{aligned} ^{C}_{0}D_{t}^{\Im }{\mathbb {L}}_2&=\left( 1- \dfrac{{S_{H}^{**}}}{{S_{H}}}\right) ~^{C}_{0}D_{t}^{\Im }{S_{H}}+\left( 1- \dfrac{{I_{1D}^{**}}}{{I_{1D}}}\right) ~^{C}_{0}D_{t}^{\Im }{I_{1D}}+\left( 1- \dfrac{{I_{2D}^{**}}}{{I_{2D}}}\right) ~^{C}_{0}D_{t}^{\Im }{I_{2D}}+\left( 1- \dfrac{{I_{C}^{**}}}{{I_{C}}}\right) ~^{C}_{0}D_{t}^{\Im }{I_{C}}\\&+\left( 1- \dfrac{{S_{V}^{**}}}{{S_{V}}}\right) ~^{C}_{0}D_{t}^{\Im }{S_{V}}+\left( 1- \dfrac{{I_{1V}^{**}}}{{I_{1V}}}\right) ~^{C}_{0}D_{t}^{\Im }{I_{1V}}+\left( 1- \dfrac{{I_{2V}^{**}}}{{I_{2V}}}\right) ~^{C}_{0}D_{t}^{\Im }{I_{2V}} \end{aligned}$$

Substituting (10) into (11) we have that,

12 $$\begin{aligned} &\left( 1-\dfrac{{S_{H}^{**}}}{{S_{H}}}\right) \left[ {\omega _{H}}-\left( \dfrac{{\beta _{1V}}{I_{1V}}}{{N_{H}}}+\dfrac{{\beta _{2V}}{I_{2V}}}{{N_{H}}}+\dfrac{{\beta _{C}}{I_{C}} }{{N_{H}}}+{\mu _{H}}\right) {S_{H}}\right] +\left( 1- \dfrac{{I_{1D}^{**}}}{{I_{1D}}}\right) \\ {}&\times \left[ \dfrac{{\beta _{1V}}{I_{1V}}}{{N_{H}}}{S_{H}}-\left( {\mu _{H}}+{\delta _{1D}}+{\uptau _{1D}}\right) {I_{1D}}\right] +\left( 1- \dfrac{{I_{2D}^{**}}}{{I_{2D}}}\right) \left[ \dfrac{{\beta _{2V}}{I_{2V}}}{{N_{H}}}{S_{H}}-\left( {\mu _{H}}+{\delta _{2D}}+{\uptau _{2D}}\right) {I_{2D}}\right] \\&\left( 1- \dfrac{{I_{C}^{**}}}{{I_{C}}}\right) \left[ \dfrac{{\beta _{C}}{I_{C}} }{{N_{H}}}{S_{H}}-\left( {\mu _{H}}+{\delta _{C}}+{\uptau _{C}}\right) {I_{C}}\right] +\left( 1- \dfrac{{S_{V}^{**}}}{{S_{V}}}\right) \left[ {\omega _{V}}-\left( \dfrac{{\beta _{1D}}{I_{1D}}}{{N_{H}}}+\dfrac{{\beta _{2D}}{I_{2D}}}{{N_{H}}}+{\mu _{V}}\right) {S_{V}}\right] \\ +&\left( 1- \dfrac{{I_{1V}^{**}}}{{I_{1V}}}\right) \left[ \dfrac{{\beta _{1D}}{I_{1D}}}{{N_{H}}}{S_{V}}-{\mu _{V}}{I_{1V}}\right] +\left( 1- \dfrac{{I_{2V}^{**}}}{{I_{2V}}}\right) \left[ \dfrac{{\beta _{2D}}{I_{2D}}}{{N_{H}}}{S_{V}}-{\mu _{V}}{I_{2V}}\right] \end{aligned}$$

From model (10) at steady state, we obtain

13 $$\begin{aligned} {\omega _{H}}&=\left( \dfrac{{\beta _{1V}}{I_{1V}^{**}}}{{N_{H}^{**}}}+\dfrac{{\beta _{2V}}{I_{2V}^{**}}}{{N_{H}^{**}}}+\dfrac{{\beta _{C}}{I_{C}^{**}}}{{N_{H}^{**}}}+{\mu _{H}}\right) {S_{H}^{**}},\quad \dfrac{{\beta _{1V}}{I_{1V}^{**}}}{{N_{H}^{**}}}{S_{H}^{**}}=q_1{I_{1D}^{**}},\quad \dfrac{{\beta _{2V}}{I_{2V}^{**}}}{{N_{H}^{**}}}{S_{H}^{**}}=q_2{I_{2D}^{**}}\\ \dfrac{{\beta _{C}}{I_{C}^{**}}}{{N_{H}^{**}}}{S_{H}^{**}}&=q_3{I_{C}^{**}},\quad {\tau _{1D}}{I_{ID}^{**}}+{\tau _{2D}}{I_{2D}^{**}}={\mu {H}}{R_{D}^{**}},\quad {\tau _{C}}{I_{C}^{**}}={\mu {H}}{R_{C}^{**}},\quad {\omega _{V}}=\left( \dfrac{{\beta _{1D}}{I_{1D}^{**}}}{{N_{H}^{**}}}+\dfrac{{\beta _{2D}}{I_{2D}^{**}}}{{N_{H}^{**}}}+{\mu _{V}}\right) {S_{V}^{**}}\\ \dfrac{{\beta _{1D}}{I_{1D}^{**}}}{{N_{H}^{**}}}{S_{V}^{**}}&={\mu _{V}}{I_{1V}^{**}},\quad \dfrac{{\beta _{2D}}{I_{2D}^{**}}}{{N_{H}^{**}}}{S_{V}^{**}}={\mu _{V}}{I_{2V}^{**}} \end{aligned}$$

Substituting equation(13) into (12), we obtain

$$\begin{aligned} ^{C}_{0}D_{t}^{\Im }{\mathbb {L}}_2&=\left( 1- \dfrac{{S_{H}^{**}}}{{S_{H}}}\right) \left[ \left( \dfrac{{\beta _{1V}}{I_{1V}^{**}}}{{N_{H}^{**}}}+\dfrac{{\beta _{2V}}{I_{2V}^{**}}}{{N_{H}^{**}}}+\dfrac{{\beta _{C}}{I_{C}^{**}}}{{N_{H}^{**}}}+{\mu _{H}}\right) {S_{H}^{**}}-\left( \dfrac{{\beta _{1V}}{I_{1V}}}{{N_{H}}}+\dfrac{{\beta _{2V}}{I_{2V}}}{{N_{H}}}+\dfrac{{\beta _{C}}{I_{C}} }{{N_{H}}}+{\mu _{H}}\right) {S_{H}}\right] +\left( 1- \dfrac{{I_{1D}^{**}}}{{I_{1D}}}\right) \\&\times \left[ \dfrac{{\beta _{1V}}{I_{1V}}}{{N_{H}}}{S_{H}}-q_1{I_{1D}}\right] +\left( 1- \dfrac{{I_{2D}^{**}}}{{I_{2D}}}\right) \left[ \dfrac{{\beta _{2V}}{I_{2V}}}{{N_{H}}}{S_{H}}-q_2{I_{2D}}\right] +\left( 1- \dfrac{{I_{C}^{**}}}{{I_{C}}}\right) \left[ \dfrac{{\beta _{C}}{I_{C}} }{{N_{H}}}{S_{H}}-q_3{I_{C}}\right] +\left( 1- \dfrac{{S_{V}^{**}}}{{S_{V}}}\right) \\&\times \left[ \left( \dfrac{{\beta _{1D}}{I_{1D}^{**}}}{{N_{H}^{**}}}+\dfrac{{\beta _{2D}}{I_{2D}^{**}}}{{N_{H}^{**}}}+{\mu _{V}}\right) {S_{V}^{**}}\right. \left. -\left( \dfrac{{\beta _{1D}}{I_{1D}}}{{N_{H}}}+\dfrac{{\beta _{2D}}{I_{2D}}}{{N_{H}}}+{\mu _{V}}\right) {S_{V}}\right] +\left( 1- \dfrac{{I_{1V}^{**}}}{{I_{1V}}}\right) \left( \dfrac{{\beta _{1D}}{I_{1D}}}{{N_{H}}}{S_{V}}-{\mu _{V}}{I_{1V}}\right) \\&+\left( 1- \dfrac{{I_{2V}^{**}}}{{I_{2V}}}\right) \left( \dfrac{{\beta _{2D}}{I_{2D}}}{{N_{H}}}{S_{V}}-{\mu _{V}}{I_{2V}}\right) \end{aligned}$$

which can be written as;

$$\begin{aligned} ^{C}_{0}D_{t}^{\Im }{\mathbb {L}}_2&\le \left( 1- \dfrac{{S_{H}^{**}}}{{S_{H}}}\right) \left[ \left( {\beta _{1V}}{I_{1V}^{**}}+{\beta _{2V}}{I_{2V}^{**}}+{\beta _{C}}{I_{C}^{**}}+{\mu _{H}}\right) {S_{H}^{**}}-\left( {\beta _{1V}}{I_{1V}}+{\beta _{2V}}{I_{2V}}+{\beta _{C}}{I_{C}}+{\mu _{H}}\right) {S_{H}}\right] +\left( 1- \dfrac{{I_{1D}^{**}}}{{I_{1D}}}\right) \\&\times \left[ {\beta _{1V}}{I_{1V}}{S_{H}}-q_1{I_{1D}}\right] +\left( 1- \dfrac{{I_{2D}^{**}}}{{I_{2D}}}\right) \left[ {\beta _{2V}}{I_{2V}}{S_{H}}-q_2{I_{2D}}\right] +\left( 1- \dfrac{{I_{C}^{**}}}{{I_{C}}}\right) \left[ {\beta _{C}}{I_{C}} {S_{H}}-q_3{I_{C}}\right] +\left( 1- \dfrac{{S_{V}^{**}}}{{S_{V}}}\right) \\&\times \left[ \left( {\beta _{1D}}{I_{1D}^{**}}+{\beta _{2D}}{I_{2D}^{**}}+{\mu _{V}}\right) {S_{V}^{**}}\right. \left. -\left( {\beta _{1D}}{I_{1D}}+{\beta _{2D}}{I_{2D}}+{\mu _{V}}\right) {S_{V}}\right] +\left( 1- \dfrac{{I_{1V}^{**}}}{{I_{1V}}}\right) \left( {\beta _{1D}}{I_{1D}}{S_{V}}-{\mu _{V}}{I_{1V}}\right) \\&+\left( 1- \dfrac{{I_{2V}^{**}}}{{I_{2V}}}\right) \left( {\beta _{2D}}{I_{2D}}{S_{V}}-{\mu _{V}}{I_{2V}}\right) \end{aligned}$$

On further simplification we have that,

$$\begin{aligned} ^{C}_{0}D_{t}^{\Im }{\mathbb {L}}_2&={\mu _{H}}{S_{H}^{**}}\left( 2-\dfrac{{S_{H}}}{{S_{H}^{**}}}-\dfrac{{S_{H}^{**}}}{{S_{H}}}\right) +{\beta _{C}}{I_{C}^{**}}{S_{H}^{**}}\left( 2-\dfrac{{S_{H}}}{{S_{H}^{**}}}-\dfrac{{S_{H}^{**}}}{{S_{H}}}\right) +{\mu _{V}}{S_{V}^{**}}\left( 2-\dfrac{{S_{V}}}{{S_{V}^{**}}}-\dfrac{{S_{V}^{**}}}{{S_{V}}}\right) \\&+{\beta _{1V}}{I_{1V}^{**}}{S_{H}^{**}}\left( 4-\dfrac{{S_{H}^{**}}}{{S_{H}}}-\dfrac{{S_{V}^{**}}}{{S_{V}}}-\dfrac{{I_{1V}}{I_{1D}^{**}}{S_{H}}}{{I_{1V}^{**}}{I_{1D}}{S_{H}^{**}}}-\dfrac{{I_{1V}^{**}}{I_{1D}}{S_{V}}}{{I_{1V}}{I_{1D}^{**}}{S_{V}^{**}}}\right) +{\beta _{2V}}{I_{2V}^{**}}{S_{H}^{**}}\left( 4-\dfrac{{S_{H}^{**}}}{{S_{H}}}-\dfrac{{S_{V}^{**}}}{{S_{V}}}-\dfrac{{I_{2V}}{I_{2D}^{**}}{S_{H}}}{{I_{2V}^{**}}{I_{2D}}{S_{H}^{**}}}-\dfrac{{I_{2V}^{**}}{I_{2D}}{S_{V}}}{{I_{2V}}{I_{2D}^{**}}{S_{V}^{**}}}\right) \end{aligned}$$

Furthermore, using the fact that geometric mean is less than arithmetic mean, we achieve the following inequalities

$$\begin{aligned} &\left( 2-\dfrac{{S_{H}}}{{S_{H}^{**}}}-\dfrac{{S_{H}^{**}}}{{S_{H}}}\right) \le 0, \quad \left( 2-\dfrac{{S_{V}}}{{S_{V}^{**}}}-\dfrac{{S_{V}^{**}}}{{S_{V}}}\right) \le 0,\quad \left( 4-\dfrac{{S_{H}^{**}}}{{S_{H}}}-\dfrac{{S_{V}^{**}}}{{S_{V}}}-\dfrac{{I_{1V}}{I_{1D}^{**}}{S_{H}}}{{I_{1V}^{**}}{I_{1D}}{S_{H}^{**}}}-\dfrac{{I_{1V}^{**}}{I_{1D}}{S_{V}}}{{I_{1V}}{I_{1D}^{**}}{S_{V}^{**}}}\right) \le 0,\\&\left( 4-\dfrac{{S_{H}^{**}}}{{S_{H}}}-\dfrac{{S_{V}^{**}}}{{S_{V}}}-\dfrac{{I_{2V}}{I_{2D}^{**}}{S_{H}}}{{I_{2V}^{**}}{I_{2D}}{S_{H}^{**}}}-\dfrac{{I_{2V}^{**}}{I_{2D}}{S_{V}}}{{I_{2V}}{I_{2D}^{**}}{S_{V}^{**}}}\right) \le 0. \end{aligned}$$

Thus, ${\mathbb {L}}_2$ is a Lyapunov function on $\mho$ such that ${\mathbb {L}}_2\le 0$ whenever ${\mathcal {R}}_0>1.$ Hence, the DPE is globally asymptotically stable whenever ${\mathcal {R}}_0>1$.

That is, in the absence of co-infection, re-infection and acquired immunity loss, every solution of model (4), with initial conditions in $\mho$, approaches unique disease-present equilibrium as *t* approaches $\infty$, whenever ${\mathcal {R}}_0>1.$ In relation to epidemiology, Theorem 4.3 implies that in the absence of co-infection, re-infection and immunity loss, the double infections of COVID-19 and dengue (both strains) will persist over the time whenever ${\mathcal {R}}_0>1.$

## Numerical scheme of the Caputo fractional model

Let $$N={N_{H}}+{N_{V}}$$, $$\omega ={\omega _{H}}+{\omega _{V}}$$ and $$\mu ={\mu _{H}}+{\mu _{V}}$$.

Then,

$$\begin{aligned} ^{C}_{0}D_{t}^{\Im }N(t)\le \omega -\mu N, \end{aligned}$$

with

14 $$\begin{aligned} N(t)\le N(0)E_{\Im }(-\mu h^{\Im })+\omega h^{\Im }E_{\Im , \Im +1}(-\mu h^{\Im }) \end{aligned}$$

where $E_{\Im , \Im +1}$ is the Mittag-Leffler function and $h=(t_{k+1}-t_k)$

Let $t_0=0<t_1<t_2<\cdot \cdot \cdot <t_N=T,$ such that $t_k=\frac{kT}{N}$ and $N \in {\mathbb {Z}}^+$.

Now, we present a Non-Standard Finite Difference (NSFD) scheme for the Caputo fractional model following the approach of^40^. For $t>0$ and $0<\Im <1$, the Caputo fractional derivative is expressed as

15 $$\begin{aligned} D^{\Im }_t\varphi (t)\vert _{t=t_{k+1}}=\frac{1}{\Gamma (1-\Im )}\sum _{i=0}^k\int _{t_k}^{t_{k+1}}\frac{d\varphi (s)}{ds}(t_{k+1}-s)^{-\Im }ds \end{aligned}$$

Upon discretizing $\frac{d\varphi (s)}{ds}$ on $[t_i, t_{i+1}]$ we have that

$$\begin{aligned} \frac{d\varphi (s)}{ds}=\frac{\varphi ^{i+1}-\varphi ^i}{\varphi (h)} \end{aligned}$$

where *h* is the mesh size and $\varphi ^i=\varphi (t_i)$.

But,

$$\begin{aligned} D^{\Im }_t\varphi (t)\vert _{t=t_{k+1}}\approx \frac{1}{\Gamma (2-\Im )}\sum \triangle _{\Im , k}^i\frac{\varphi ^{i+1}-\varphi ^i}{\varphi (h)} \end{aligned}$$

with

$$\begin{aligned} \triangle _{\Im , k}^i=\left( (t_{k+1}-t_i)^{1-\Im }-(t_{k+1}-t_{i+1})^{1-\Im } \right) \end{aligned}$$

If $$i=k$$ then,

16 $$\begin{aligned} \triangle _{\Im , k}^k=\left( (t_{k+1}-t_k)^{1-\Im }-(t_{k+1}-t_{k+1})^{1-\Im } \right) =(t_{k+1}-t_k)^{1-\Im }=h^{1-\Im } \end{aligned}$$

When $t=t_{k+1}$ then we obtain

17 $$\begin{aligned} \frac{1}{\Gamma (2-\Im )}\sum _{i=0}^k\triangle ^i_{\Im , k}\frac{\varphi ^{i+1}-\varphi ^i}{\psi (h)}=\zeta (\varphi ^{k+1}), ~k=1, 2, \cdot \cdot \cdot , K-1 \end{aligned}$$

Applying the NSFD scheme (17) to the inequality (14) we have that

18 $$\begin{aligned} \frac{1}{\Gamma (2-\Im )}\sum _{i=0}^k\triangle ^i_{\Im , k}\frac{(N^{i+1}-N^i)}{\psi (h)}\le \omega -\mu N^{k+1} \end{aligned}$$

at $i=k$, we have that

19 $$\begin{aligned} \frac{h^{1-\Im }}{\Gamma (2-\Im )}\frac{(N^{k+1}-N^k)}{\psi (h)}+\sum _{i=0}^{k-1}\triangle _{\Im , k}^i(N^{i+1}-N^i)\le \Gamma (2-\Im )\psi (h)(\omega -\mu N^{k+1}) \end{aligned}$$

Now,

20 $$\begin{aligned} N^{k+1}\left( h^{1-\Im }+\mu \Gamma (2-\Im )\psi (h)\right) \le h^{1-\Im }N^k+\Gamma (2-\Im )\psi (h)\omega -\sum _{i=0}^{k-1}\triangle _{\Im , k}^i(N^{i+1}-N^i) \end{aligned}$$

Thus,

21 $$\begin{aligned} N^{k+1}\le \frac{h^{1-\Im }N^k+\omega \Gamma (2-\Im )\psi (h)-\sum _{i=0}^{k-1}\triangle _{\Im , k}^i(N^{i+1}-N^i)}{h^{1-\Im }+\mu \Gamma (2-\Im )\psi (h)} \end{aligned}$$

When $k=0$, we have that

22 $$\begin{aligned} N^1\le \left( \frac{h^{1-\Im }N^0}{h^{1-\Im }+\mu \Gamma (2-\Im )\psi (h)}+\frac{\omega \Gamma (2-\Im )\psi (h)}{h^{1-\Im }+\mu \Gamma (2-\Im )\psi (h)}\right) \end{aligned}$$

To determine the denominator function $(\psi (h))$, we compare equations (14) and (22)

Hence,

$$\begin{aligned} \psi (h)=\frac{h^{1-\Im }\left( 1-E_{\Im }(-\mu h^{\Im })\right) }{\mu \Gamma (2-\Im )E_{\Im }(-\mu h^{\Im })} \end{aligned}$$

Next, we apply the NSFD scheme on model (4) and we obtain the following difference equations

23 $$\begin{aligned} {S_{H}^{k+1}}&= \frac{h^{1-\Im }{S_{H}^k}+\Gamma (2-\Im )\psi (h)\left( {\omega _{H}}+{\theta _{D}}{R_{D}^{k}}+{\theta _{C}}{R_{C}^{k}}\right) -\sum _{i=0}^{k-1}\triangle ^i_{\Im , k}({S_{H}^{i+1}}-{S_{H}^i})}{\Gamma (2-\Im )\psi (h)\left[ \frac{{\beta _{1V}}{I_{1V}^k}}{{N_{H}^k}} +\frac{{\beta _{2V}}{I_{2V}^k}}{{N_{H}^k}}+\frac{{\beta _{C}}\left( {I_{C}^k}+{I_{1DC}^k}+{I_{2DC}^k}\right) }{{N_{H}^k}}+{\mu _{H}}\right] +h^{1-\Im }}\\ {I_{1D}^{k+1}}&= \frac{h^{1-\Im }{I_{1D}^k}+\Gamma (2-\Im )\psi (h)\left[ \frac{{\beta _{1V}}{I_{1V}^k}}{{N_{H}^k}}\left( {S_{H}^{k+1}}+{R_{C}^{k}}\right) +{\uptau _{C}}{I_{1DC}^{k}}\right] -\sum _{i=0}^{k-1}\triangle ^i_{\Im , k}({I_{1D}^{i+1}}-{I_{1D}^i})}{\Gamma (2-\Im )\psi (h)\left[ {\mu _{H}}+{\delta _{1D}}+{\uptau _{1D}}+\frac{{\varepsilon _{C}}{\beta _{C}}\left( {I_{C}^k}+{I_{1DC}^k}+{I_{2DC}^k}\right) }{{N_{H}^k}}\right] +h^{1-\Im }}\\ {I_{2D}^{k+1}}&= \frac{h^{1-\Im }{I_{2D}^k}+\Gamma (2-\Im )\psi (h)\left[ \frac{{\beta _{2V}}{I_{2V}^k}}{{N_{H}^k}}\left( {S_{H}^{k+1}}+{R_{C}^{k}}\right) +{\uptau _{C}}{I_{2DC}^{k}}\right] -\sum _{i=0}^{k-1}\triangle ^i_{\Im , k}({I_{2D}^{i+1}}-{I_{2D}^i})}{\Gamma (2-\Im )\psi (h)\left[ {\mu _{H}}+{\delta _{2D}}+{\uptau _{2D}}+\frac{{\varepsilon _{C}}{\beta _{C}}\left( {I_{C}^k}+{I_{1DC}^k}+{I_{2DC}^k}\right) }{{N_{H}^k}}\right] +h^{1-\Im }}\\ {I_{C}^{k+1}}&= \frac{h^{1-\Im }{I_{C}^k}+\Gamma (2-\Im )\psi (h)\left[ \frac{{\beta _{C}}\left( {I_{C}^k}+{I_{1DC}^k}+{I_{2DC}^k}\right) }{{N_{H}^k}}\left( {S_{H}^{k+1}}+{R_{D}^{k}}\right) +{\uptau _{1D}}{I_{1DC}^{k}}+{\uptau _{2D}}{I_{2DC}^{k}}\right] -\sum _{i=0}^{k-1}\triangle ^i_{\Im , k}({I_{C}^{i+1}}-{I_{C}^i})}{\Gamma (2-\Im )\psi (h)\left[ {\mu _{h}}+{\delta _{C}}+{\uptau _{C}}+\left( \frac{{\varepsilon _{1V}}{\beta _{1V}}{I_{1V}^k}}{{N_{H}^k}}+\frac{{\varepsilon _{2V}}{\beta _{2V}}{I_{2V}^k}}{{N_{H}^k}}\right) \right] +h^{1-\Im }}\\ {I_{1DC}^{k+1}}&= \frac{h^{1-\Im }{I_{1DC}^k}+\Gamma (2-\Im )\psi (h)\left[ \frac{{\varepsilon _{C}}{\beta _{C}}\left( {I_{C}^{k+1}}+{I_{1DC}^k}+{I_{2DC}^k}\right) {I_{1D}^{k+1}}}{{N_{H}^k}}+\frac{{\varepsilon _{1V}}{\beta _{1V}}{I_{1V}^k}{I_{C}^{k+1}}}{{N_{H}^k}}\right] -\sum _{i=0}^{k-1}\triangle ^i_{\Im , k}({I_{1DC}^{i+1}}-{I_{IDC}^i})}{\Gamma (2-\Im )\psi (h)\left( {\mu _{h}}+{\delta _{1D}}+{\delta _{C}}+{\uptau _{1D}}+{\uptau _{C}}\right) +h^{1-\Im }}\\ {I_{2DC}^{k+1}}&= \frac{h^{1-\Im }{I_{2DC}^k}+\Gamma (2-\Im )\psi (h)\left[ \frac{{\varepsilon _{C}}{\beta _{C}}\left( {I_{C}^{k+1}}+{I_{1DC}^k}+{I_{2DC}^{k+1}}\right) {I_{2D}^{k+1}}}{{N_{H}^k}}+\frac{{\varepsilon _{2V}}{\beta _{2V}}{I_{2V}^k}{I_{C}^{k+1}}}{{N_{H}^k}}\right] -\sum _{i=0}^{k-1}\triangle ^i_{\Im , k}({I_{2DC}^{i+1}}-{I_{2DC}^i})}{\Gamma (2-\Im )\psi (h)\left( {\mu _{h}}+{\delta _{2D}}+{\delta _{C}}+{\uptau _{2D}}+{\uptau _{C}}\right) +h^{1-\Im }}\\ {R_{D}^{k+1}}&= \frac{h^{1-\Im }{R_{D}^k}+\Gamma (2-\Im )\psi (h)\left[ {\uptau _{1D}}{I_{1D}^{k+1}}+{\uptau _{2D}}{I_{2D}^{k+1}}\right] -\sum _{i=0}^{k-1}\triangle ^i_{\Im , k}({R_{D}^{i+1}}-{R_{D}^i})}{\Gamma (2-\Im )\psi (h)\left[ \frac{{\beta _{C}}\left( {I_{C}^k}+{I_{1DC}^k}+{I_{2DC}^k}\right) }{{N_{H}^k}}+{\mu _{H}}+{\theta _{D}}\right] +h^{1-\Im }}\\ {R_{C}^{k+1}}&= \frac{h^{1-\Im }{R_{C}^k}+\Gamma (2-\Im )\psi (h){\uptau _{C}}{I_{C}^{k+1}}-\sum _{i=0}^{k-1}\triangle ^i_{\Im , k}({R_{C}^{i+1}}-{R_{C}^i})}{\Gamma (2-\Im )\psi (h)\left[ \left( \frac{{\beta _{1V}}{I_{1V}^k}}{{N_{H}^k}}+\frac{{\beta _{2V}}{I_{2V}^k}}{{N_{H}^k}}\right) +{\mu _{H}}+{\theta _{C}}\right] +h^{1-\Im }}\\ {S_{V}^{k+1}}&= \frac{h^{1-\Im }{S_{V}^k}+\Gamma (2-\Im )\psi (h){\omega _{V}}-\sum _{i=0}^{k-1}\triangle ^i_{\Im , k}({S_{V}^{i+1}}-{S_{V}^i})}{\Gamma (2-\Im )\psi (h)\left[ \frac{{\beta _{1D}}\left( {I_{1D}^k}+{I_{1DC}^k}\right) }{{N_{H}^k}}+\frac{{\beta _{2D}}\left( {I_{2D}^k}+{I_{2DC}^k}\right) }{{N_{H}^k}}+{\mu _{V}}\right] +h^{1-\Im }}\\ {I_{1V}^{k+1}}&= \frac{h^{1-\Im }{I_{1V}^k}+\Gamma (2-\Im )\psi (h)\frac{{\beta _{1D}}\left( {I_{1D}^{k+1}}+{I_{1DC}^{k+1}}\right) }{{N_{H}^k}}{S_{V}^{k+1}}-\sum _{i=0}^{k-1}\triangle ^i_{\Im , k}({I_{1V}^{i+1}}-{I_{1V}^i})}{\Gamma (2-\Im )\psi (h){\mu _{V}}+h^{1-\Im }}\\ {I_{2V}^{k+1}}&= \frac{h^{1-\Im }{I_{2V}^k}+\Gamma (2-\Im )\psi (h)\frac{{\beta _{2D}}\left( {I_{2D}^{k+1}}+{I_{2DC}^{k+1}}\right) }{{N_{H}^k}}{S_{V}^{k+1}}-\sum _{i=0}^{k-1}\triangle ^i_{\Im , k}({I_{2V}^{i+1}}-{I_{2V}^i})}{\Gamma (2-\Im )\psi (h){\mu _{V}}+h^{1-\Im }}\\ \end{aligned}$$

## Numerical simulations

### Model fitting to real data

**Figure 4:**
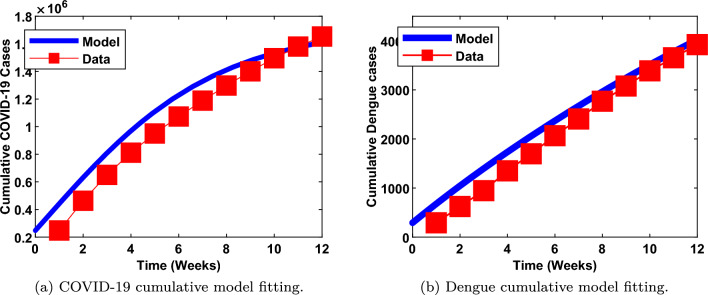
Cumulative model fittings of COVID-19 and dengue using Amazonas, Brazil data.

Demographic data^59^ of Amazonas state, Brazil is employed for the numerical study.The estimated population of the Brazilian state is 4,269,995 with life expectancy of 74.90 years^59^. Hence, the daily recruitment rate and natural human death rate are $\frac{4,269,995}{74.90\times 365}$ and $\frac{1}{74.90\times 365}$ respectively. Th initial conditions of system (4) are set as follows:$S_H(0)=3,600,000, I_{1D}(0)=145, I_{2D}(0)=146, I_C(0)=247,534, I_{1DC}(0)=0,I_{2DC}(0)=0, R_D(0)=0, R_C(0)=0, S_V(0)=100,000, I_{1V}(0)=5,000$ and $I_{2V}(0)=5,000$. Fitting of the model to real data was conducted using the MATLAB fmincon optimization algorithm. We relied on the data of Amazonas state, Brazil for COVID-19^66^ and dengue^67^ curve fittings. Based on the data, we cumulated COVID-19 and dengue active cases for a period of twelve weeks (between February and April, 2021). Within this period, it was observed that, there was a rise in combined incidences of COVID-19 and arboviruses. The estimated parameters and others from the literature, are detailed in Table (). Furthermore, model fitting to the real data are demonstrated in Fig. 4a and b. From the figures, it is clearly shown that, our model has a good fit to the real data.

### Comparison of NSFD and ODE45 solver

**Figure 5:**
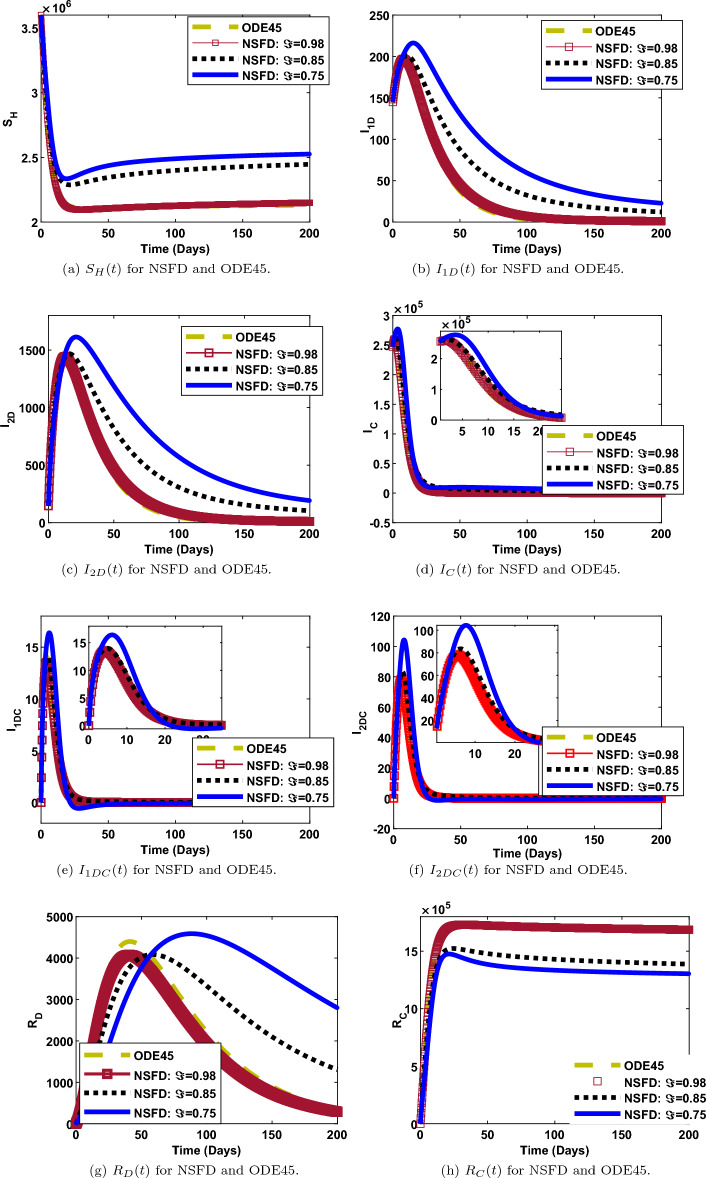
Humans for comparing NSFD and ODE45 methods.

**Figure 6:**
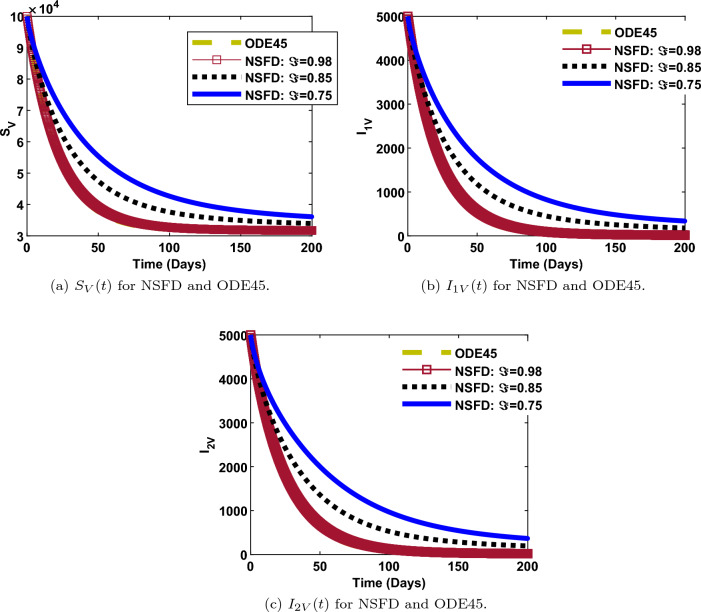
Vectors for comparing NSFD and ODE45 methods.

The numerical simulation for comparing the solutions of NSFD and ODE45 is performed at different values of the fractional-order, $\Im$. This is done for each of the disease compartment as shown in Figs. 5 and 6. It is observed that, the solutions of the NSFD scheme are sufficiently close to solutions of the ODE45 solver whenever the fractional-order is close to one. In other words, the NSFD scheme is dynamically-conformable with ODE45 when the fractional-order ($\Im =0.98$) approaches one. This is expected since ODE45 can be seen as a numerical solution to fractional-order one. On the contrary, when the NSFD scheme is simulated against fractional values ($\Im =0.85, 0.75$), it is observed that the corresponding curves differ from the curves obtained from ODE45. It is further observed that, the NSFD scheme is superior to the ODE45 solver since it shows the correct dynamic behavior of the model at different fractional values. Epidemiologically, a faster decay in the evolution of infections is observed at lower fractional values. Reverse is the case when the fractional value is higher.

**Figure 7:**
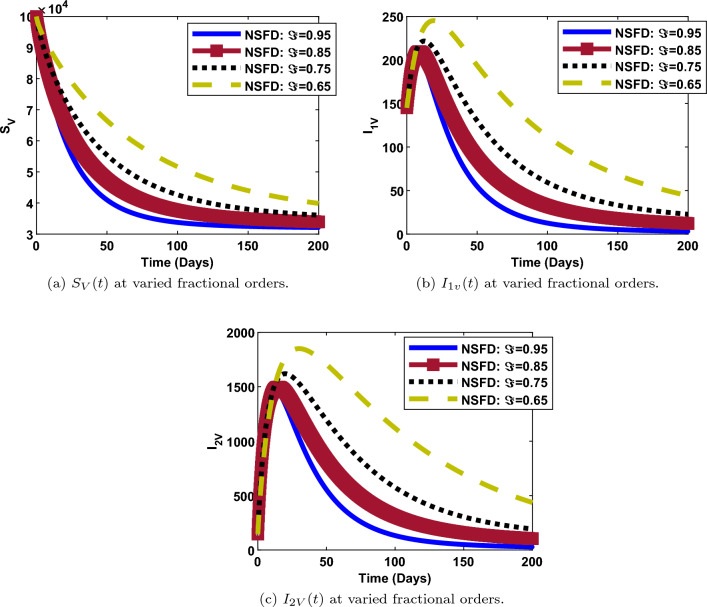
Vector populations at varied fractional orders and when $R_0=max\lbrace R_{01D},R_{02D},R_{0C}\rbrace =max\lbrace 0.00095116,0.2180,0.4887\rbrace < 1$.

### Impact of fractional order on the dynamics of each compartment

**Figure 8:**
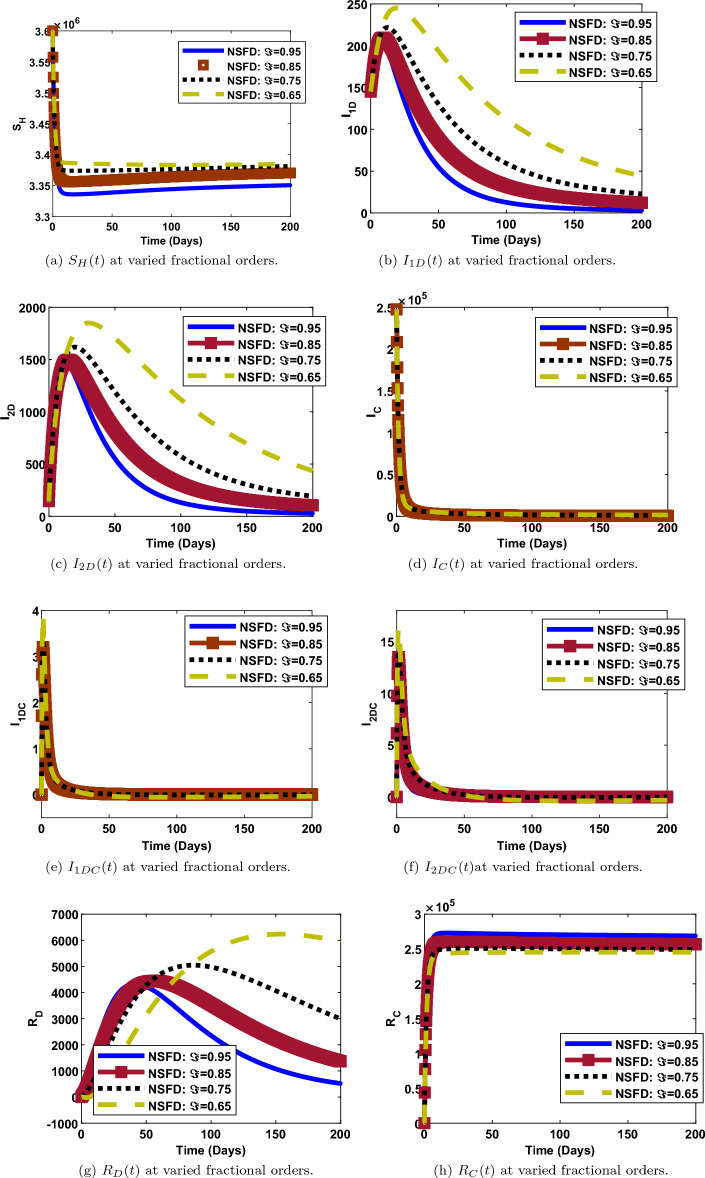
Human populations at varied fractional orders and when $R_0=max\lbrace R_{01D},R_{02D},R_{0C}\rbrace =max\lbrace 0.00095116,0.2180,0.4887\rbrace < 1$.

The numerical simulations and solution curves at varying fractional-orders $(\Im :0.95, 0.85,0.75, 0.65)$ are presented in Figs. 7 and 8 when ${\mathcal {R}}_0<1.$ Figure 8 depicts the solution curves for the human components at different fractional-orders when ${\mathcal {R}}_0<1.$ From the perspective of epidemiology, the population of susceptible individuals decays fast and stabilizes at about 3,350,000 over a period of 200 days. This happens when the fractional-order is 0.95 (close to 1). Also, it can be observed from Fig. 8a that, when $\Im$ is as low as 0.65, the population of susceptible individuals decreases slowly and stabilizes at a higher value of about 3,400,000. As depicted in Fig. 8b and c, when $\Im =0.95$ (maximum), the populations of human-infected dengue strain one and strain two decay fast from a maximum value of about 200 and 1500 respectively, to a minimum value of about 0, over a period of 200 days. However, when $\Im =0.65$ (minimum), both populations decay slowly, from a maximum value of about 250 and 1800, to a minimum value of about 50 and 500 respectively, over the same period of time. Thus, less people get infected with dengue strain one and two as the fractional-order gets close to one and vice versa. Similar trends are observed in the populations of ${I_{C}}, ~{I_{1DC}}, ~{I_{2DC}}$ though, with less impact of fractional-orders. There is something remarkable in the dynamics of recovered individuals. As observed in Fig. 8g, the number of individuals that recovered from dengue (strain one or strain two) decreases from about 4000 to 500 when $\Im =0.95$ and,increases from 0 to 6000 individuals, when $\Im$ is as low as 0.65. However, there is a direct relationship between the value of the fractional-order and, COVID19-recovered persons. Thus, more people recover from either dengue strain one or strain two for less fractional values when ${\mathcal {R}}_0<1$. A contrary scenario is the case for individuals recovered from COVID-19.

Furthermore, it can be shown in Fig. 7 that, the populations of the vector components $\left( {S_{V}},~ {I_{1V}},~{I_{2V}}\right)$ at any given time is inversely proportional to the fractional-order. That is, the higher the fractional-order, the faster the decay and vice versa.

**Figure 9:**
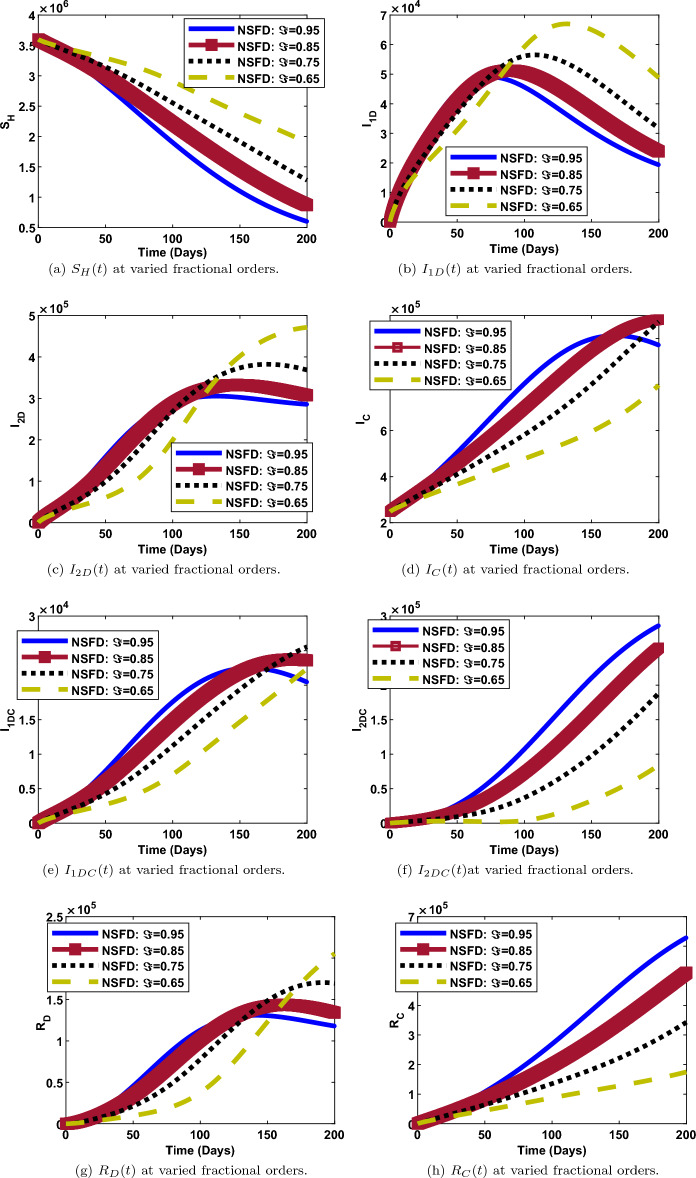
Human populations at varied fractional orders and when $R_0=max\lbrace R_{01D},R_{02D},R_{0C}\rbrace =max\lbrace 1.4456,2.1152,2.6784\rbrace > 1$.

**Figure 10:**
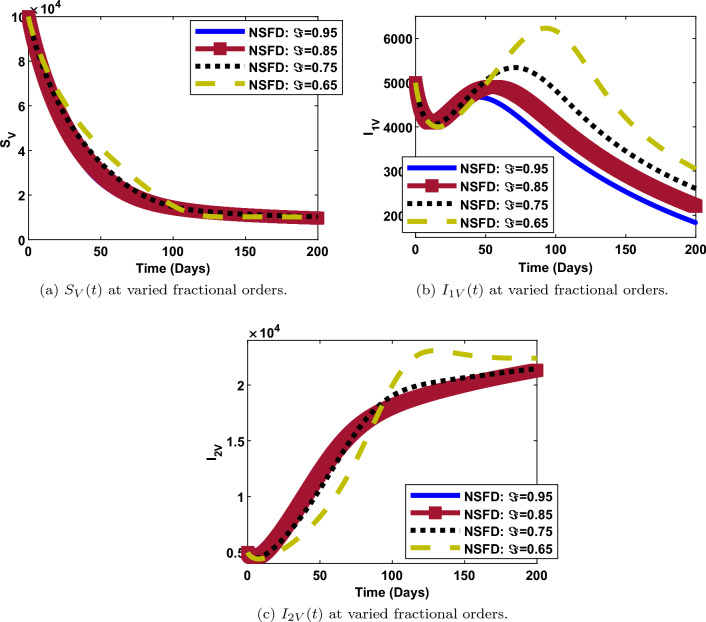
Vector populations at varied fractional orders and when $R_0=max\lbrace R_{01D},R_{02D},R_{0C}\rbrace =max\lbrace 1.3737,1.5985,2.1152\rbrace > 1$.

On the other hand, we examined the impact of fractional-order, $(\Im )$ on the trajectories of human and vector components when ${\mathcal {R}}_0>1$.This is depicted in Figs. 9 and 10. The simulation is for a period of 200 days with $\Im$ ranging from 0.65 to 0.95. It can be observed that the fractional-order has a significant impact in the dynamics of both components as observed in the solution curves. From Figs. 9b,c, 10b and c, it can be observed that, the number of individuals and vectors infected with both strains of dengue, decreases from its peak to the lowest number, as $\Im$ increases from 0.65 to 0.95. The same observation is made for the populations of susceptible humans, susceptible vectors and dengue-recovered individuals as shown in Figs. 9a, 10a and 9f respectively. Reverse situation is observed for the populations of COVID19-infected individuals, co-infected individuals and individuals recovered from COVID-19. This is presented in Figs. 9d, e and g. Thus, with respect to epidemiology, the burden of dengue and COVID-19 infections can be better managed with a good knowledge of fractional-order.

### Numerical experiment of the reproduction number

**Figure 11:**
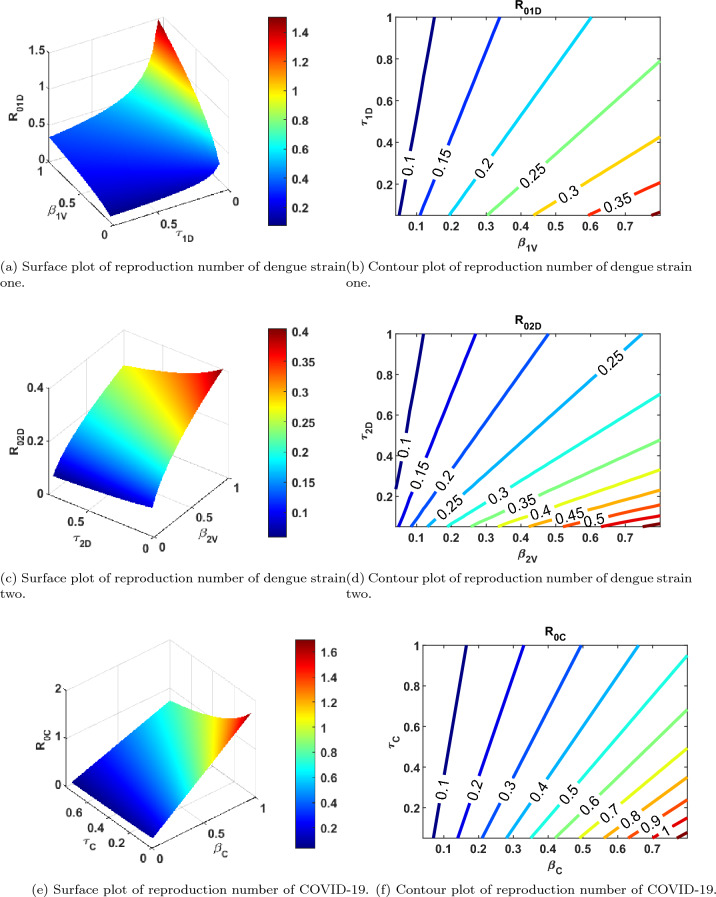
Surface and Contour plots of respective reproduction numbers as a function of transmission and recovery rates.

In this section, we present the numerical experiment of the associated reproduction numbers, as a response function to the disease transmission and recovery rates. This is illustrated in Fig. 11. It can be observed from the respective Fig. 11a–f that, the value of the associated reproduction numbers depends on the disease transmission and recovery rates. That is, an increase in transmission rates of dengue strain one, strain two and COVID-19, will result to an increase in the respective reproduction numbers. Conversely, an increase in recovery rates of dengue strain one, strain two and COVID-19, will result to a decrease in the corresponding reproduction numbers and vice versa. Epidemiologically, the co-infection or single infections of dengue (both strain one and strain two) and COVID-19 can be abated if the transmission rates are adequately low. More so, the burden of dengue and COVID-19 infections can be reduced when the recovery rates are adequately high. This may be achieved through enhanced recovery strategies and interventions.

Similarly, numerical experiments of the respective reproduction numbers as response functions of transmission and recovery rates of dengue strain one $({\beta _{1V}}, {\tau _{1D}})$, dengue strain two $({\beta _{2V}}, {\tau _{2D}})$ and COVID-19 $({\beta _{C}}, {\tau _{C}})$ are demonstrated in Fig. 11b, d and f, using contour plots. It can be equally observed that low transmission rates and high recovery rates will result to decrease in the associated reproduction numbers. This affirms that with low transmission rates and high recovery rates, dengue strain one, dengue strain two and COVID-19 can be curtailed within the population.

### Phase portraits at different initial conditions and fractional orders

**Figure 12:**
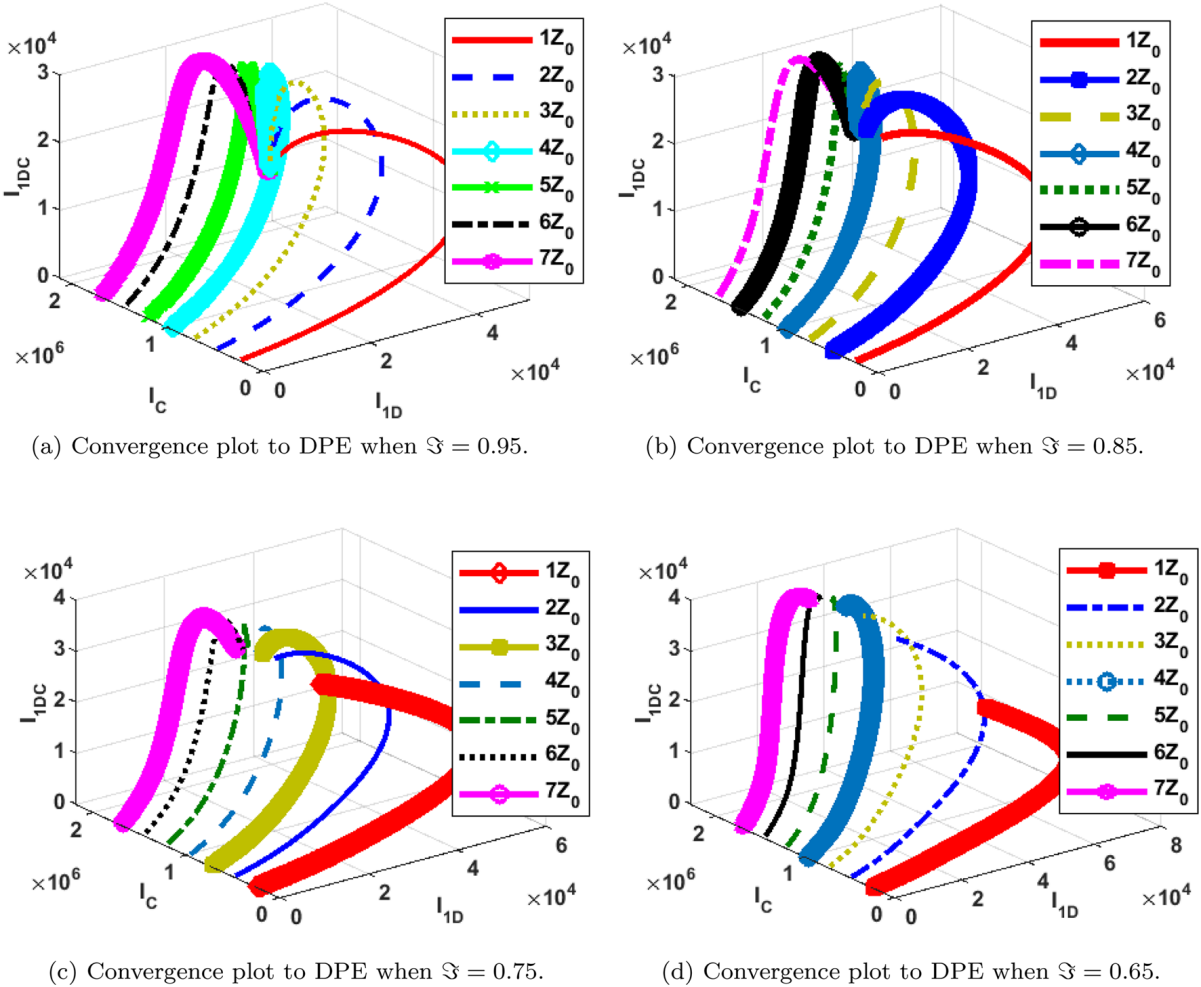
Phase portrait of $I_{1DC}, I_C$ and $I_{1D}$ showing convergence to DPE at varying fractional orders when $R_0= max\lbrace R_{01D}, R_{02D}, R_{0C}\rbrace =max \lbrace 1.4456,2.1152,2.6784\rbrace >1$, with $Z_0=\lbrace I_{1DC}(0)=0, I_C(0)=247,534, I_{1D}(0)=145\rbrace$.

**Figure 13:**
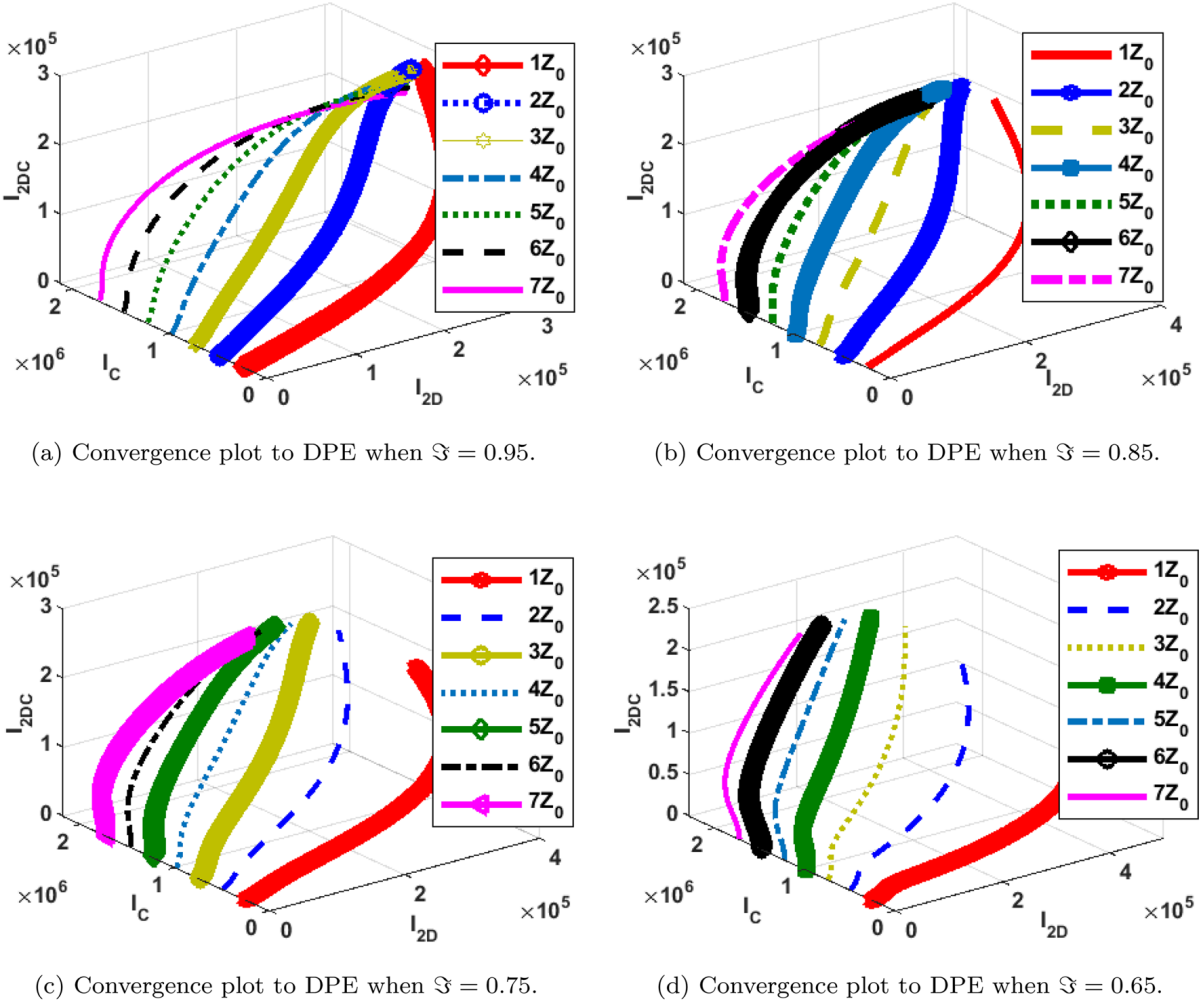
Phase portrait of $I_{2DC}, I_C$ and $I_{2D}$ showing convergence to DPE at varying fractional orders when $R_0= max\lbrace R_{01D}, R_{02D}, R_{0C}\rbrace =max \lbrace 1.4456,2.1152,2.6784\rbrace >1$, with $Z_0=\lbrace I_{2DC}(0)=0,I_C(0)= 247,534, I_{2D}(0)=146\rbrace$.

**Figure 14:**
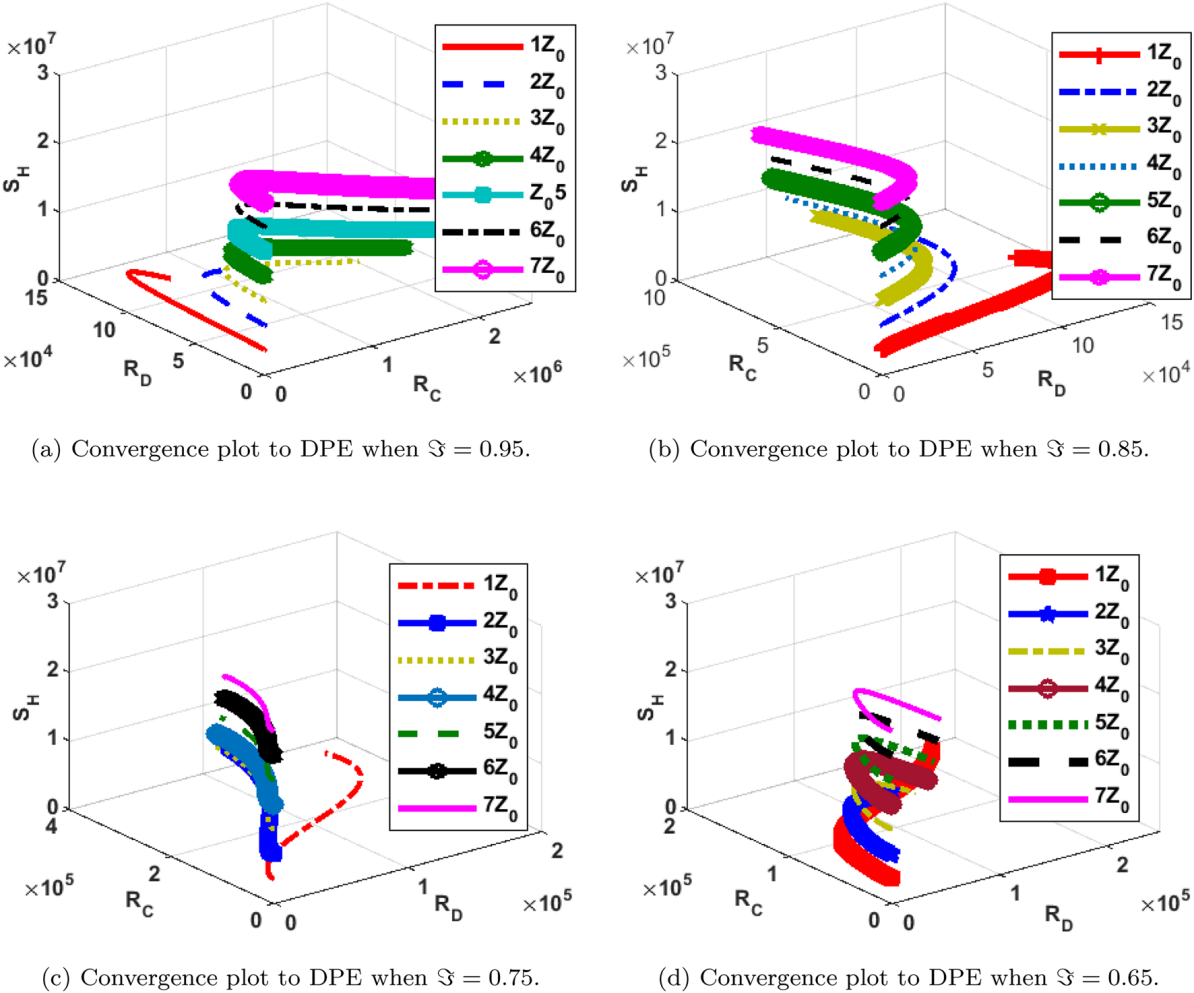
Phase portrait of $S_H, R_{D}$ and $R_{C}$ showing convergence to DPE at varying fractional orders when $R_0= max\lbrace R_{01D}, R_{02D}, R_{0C}\rbrace =max \lbrace 1.4456,2.1152,2.6784\rbrace >1$, with $Z_0=\lbrace S_H(0)=3,600,000, R_{D}(0)=0, R_{C}(0)=0\rbrace$.

**Figure 15:**
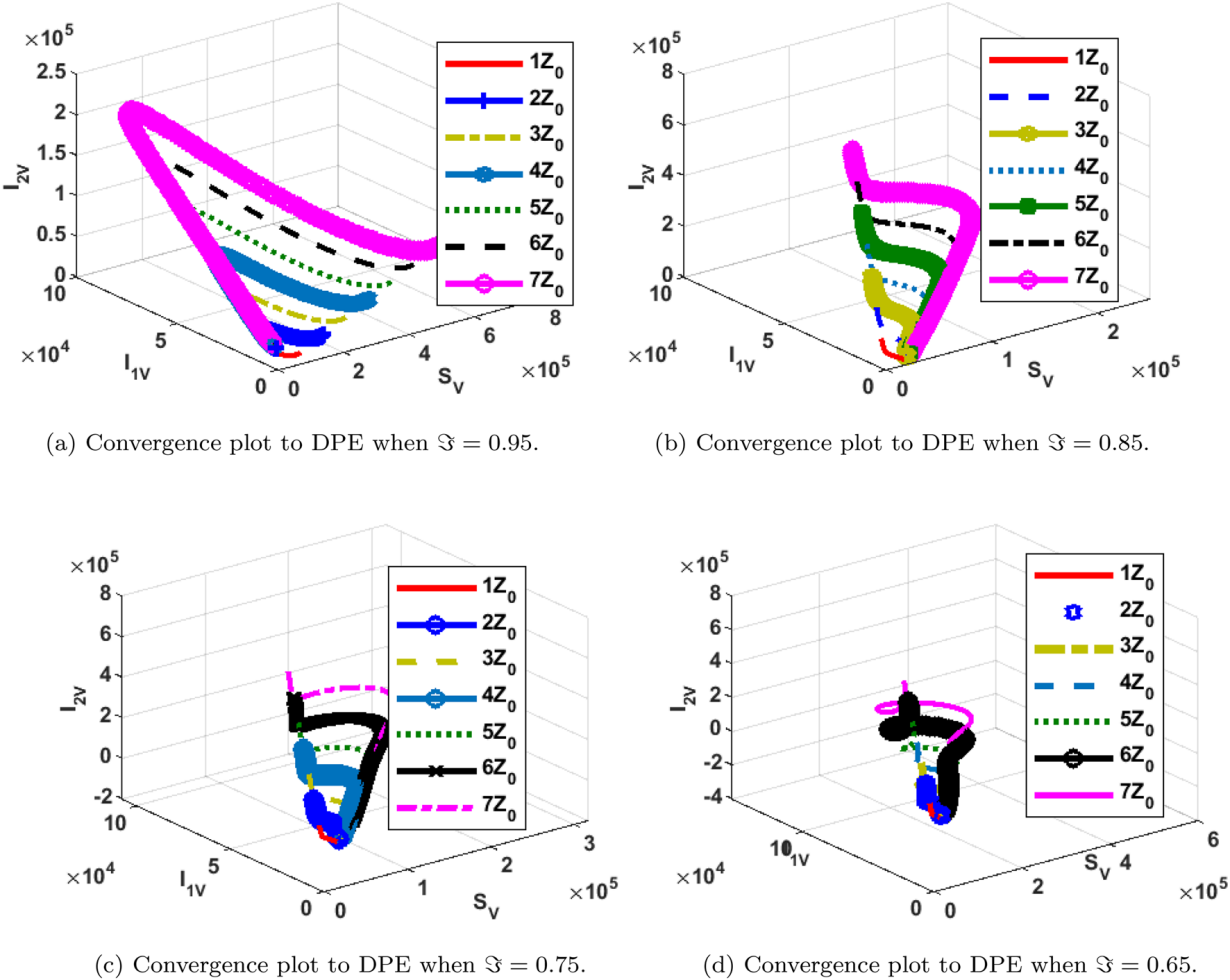
Phase portrait of $I_{2V}, S_V$ and $I_{1V}$ showing convergence to DPE at varying fractional orders when $R_0= max\lbrace R_{01D}, R_{02D}, R_{0C}\rbrace =max \lbrace 1.4456,2.1152,2.6784\rbrace >1$, with $Z_0=\lbrace I_{2V}(0)=5000, I_{1V}(0)=5000 S_V(0)=100,000 \rbrace$.

**Figure 16:**
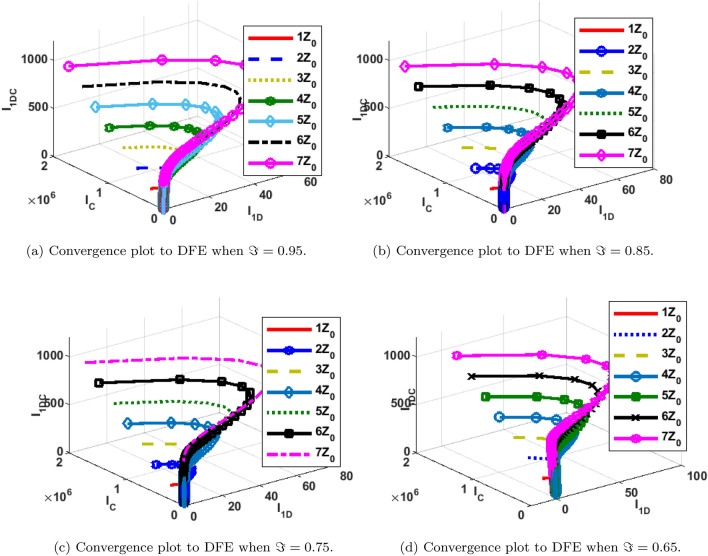
Phase portrait of $I_{1DC}, I_C$ and $I_{1D}$ showing convergence to DPE at varying fractional orders when $R_0=max\lbrace R_{01D}, R_{02D}, R_{0C}\rbrace = max \lbrace 0.0737, 0.2180, 0.5293\rbrace <1$, with $Z_0=\lbrace I_{1DC}(0)=0, I_C(0)=247,534, I_{1D}(0)=145\rbrace$.

**Figure 17:**
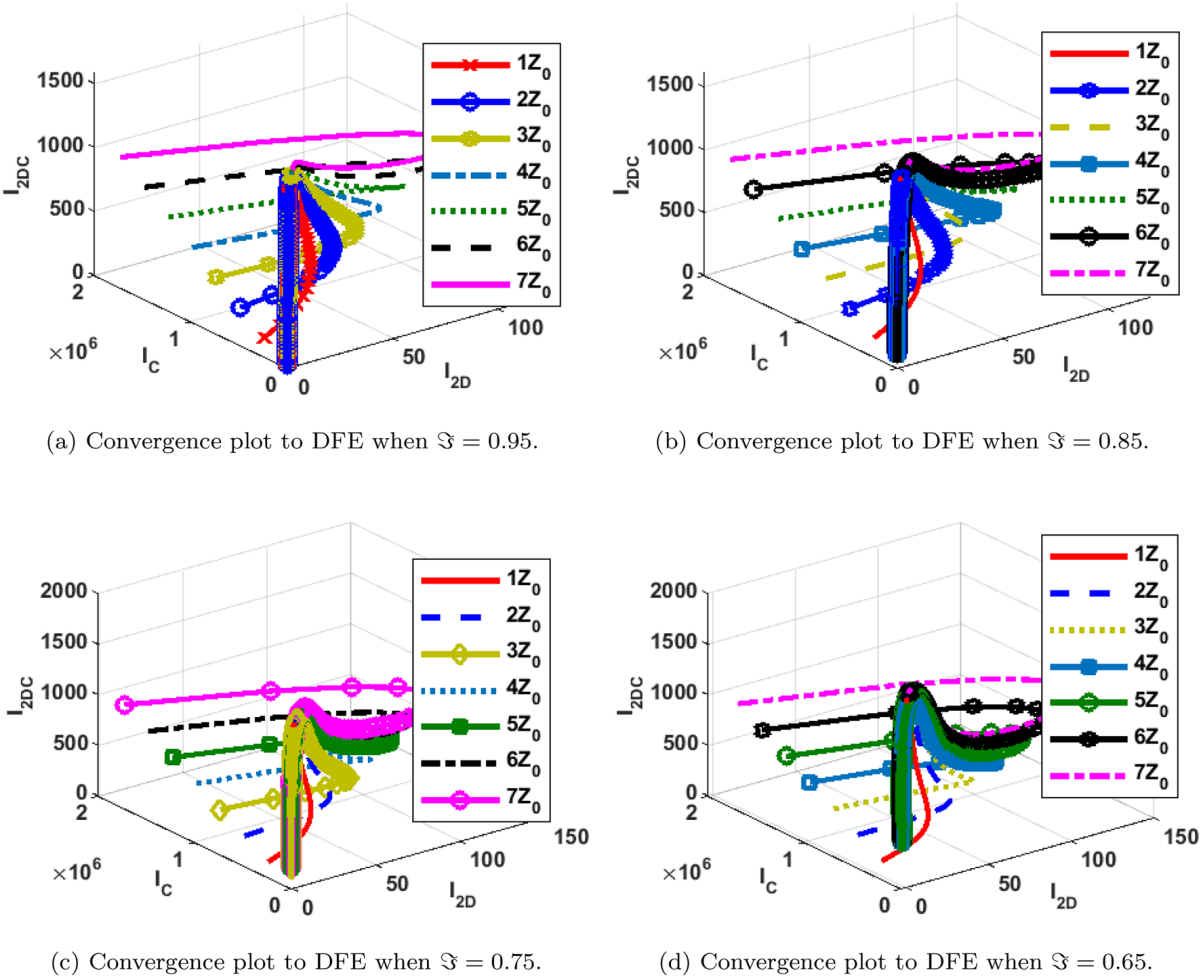
Phase portrait of $I_{2DC}, I_C$ and $I_{2D}$ showing convergence to DPE at varying fractional orders when $R_0=max\lbrace R_{01D}, R_{02D}, R_{0C}\rbrace = max \lbrace 0.0737, 0.2180, 0.5293\rbrace <1$, with $Z_0=\lbrace I_{2DC}(0)=0, I_C(0)=247,534,146\rbrace$.

**Figure 18:**
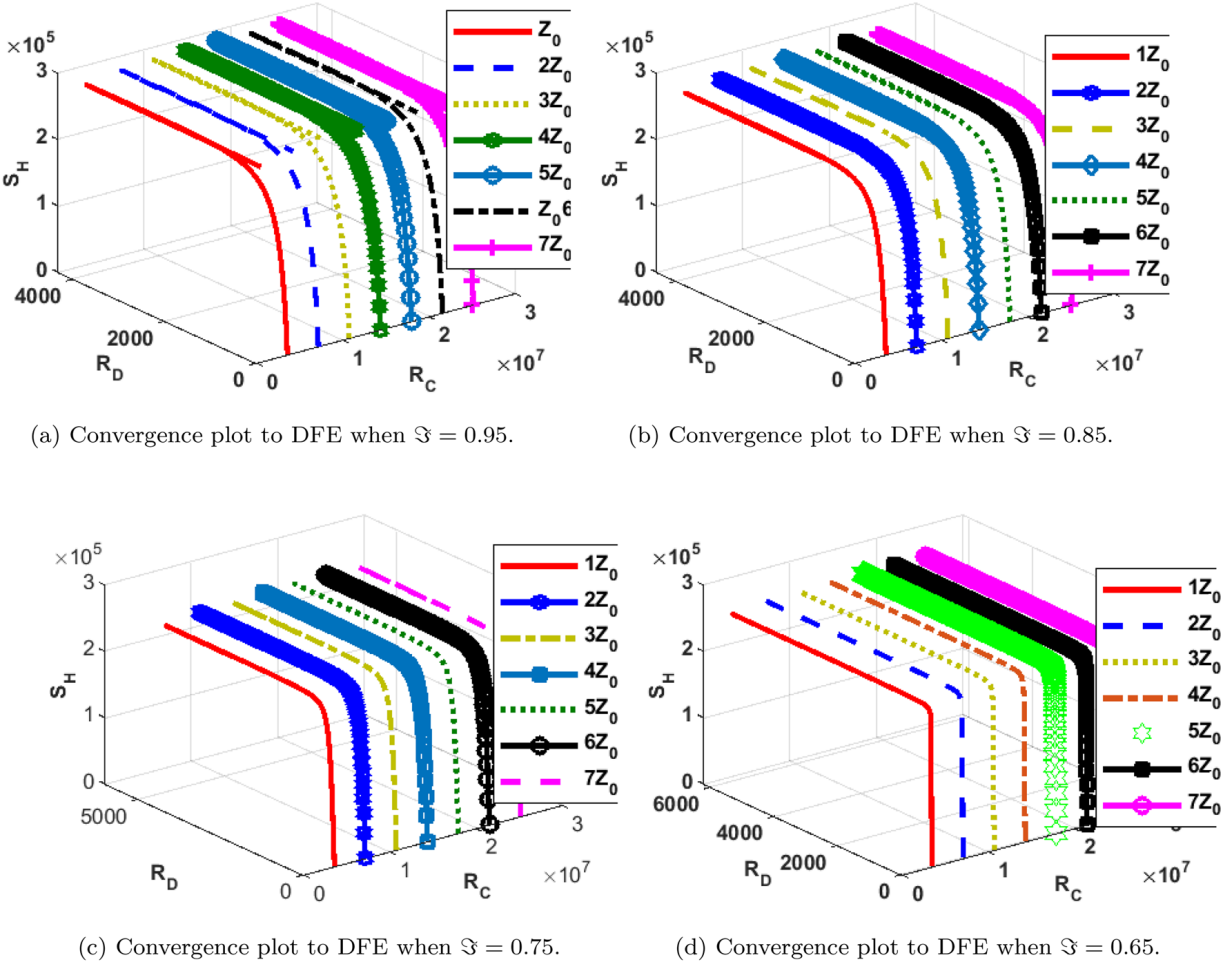
Phase portrait of $S_H, R_{D}$ and $R_{C}$ showing convergence to DPE at varying fractional orders when $R_0=max\lbrace R_{01D}, R_{02D}, R_{0C}\rbrace = max \lbrace 0.0737, 0.2180, 0.5293\rbrace <1$, with $Z_0=\lbrace S_H(0)=3,600,000, R_{D}(0)=0, R_{C}(0)=0\rbrace$.

**Figure 19:**
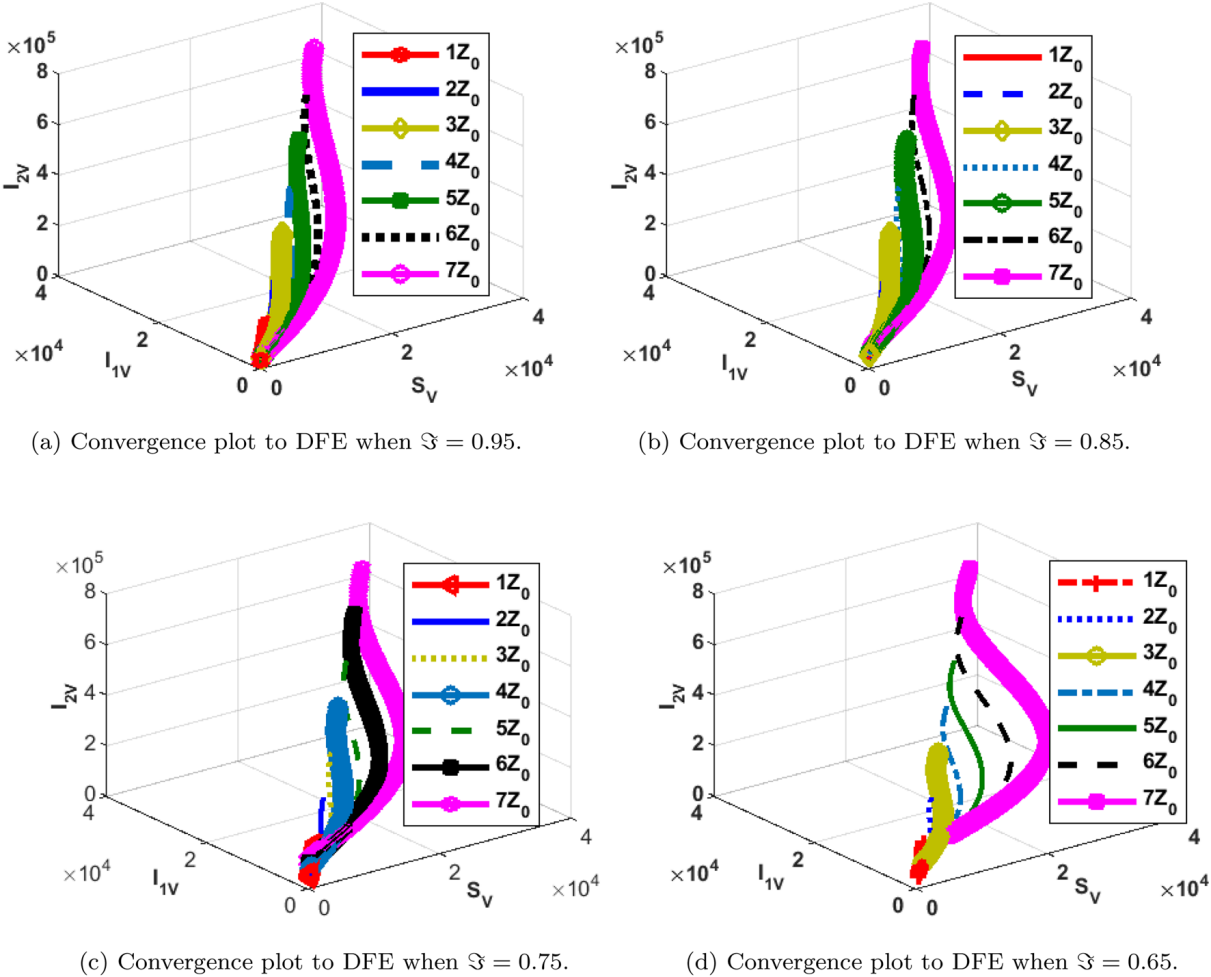
Phase portrait of $I_{2V}, S_V$ and $I_{1V}$ showing convergence to DPE at varying fractional orders when $R_0=max\lbrace R_{01D}, R_{02D}, R_{0C}\rbrace = max \lbrace 0.0737, 0.2180, 0.5293\rbrace <1$, with $Z_0=\lbrace I_{2V}(0)=5000, I_{1V}(0)=5000 S_V(0)=100,000, \rbrace$.

The phase portrait/ convergence plot to the stable disease equilibria is presented in this section “Phase portraits at different initial conditions and fractional orders”. This is for different cases of the reproduction number, ${\mathcal {R}}_0$, different values of the fractional-order and initial conditions. It can be observed from Figs. 12, 13, 14, 15, 16, 17, 18 and 19 that, all the trajectories for the human and vector components approaches the DFE when ${\mathcal {R}}_0 <1$. This is a validation of Theorems 4.1 and 4.2 respectively. Hence, the solution curves are stable and approach the DFE. Though, the convergence is independent of the changes in fractional-order, it may be observed from the plots, that the human trajectories appear to have a shorter path to the DFE as $\Im$ decreases. Thus, suggesting that the human trajectories tends to the DFE faster with decreasing values of $\Im .$ However, it is observed that the trajectories of the vector components have a different scenario in that direction. That is, slower convergence is observed for lesser values of $\Im$. Also, with different assumed initial conditions for the state variables, the solution curves tend towards the DFE over time. Hence, from an epidemiological viewpoint, the disease-free human population can be reached when ${\mathcal {R}}_0<1$ and faster when $\Im$ is not close to one. Also, the disease-free vector population can be reached when ${\mathcal {R}}_0<1$ and faster when $\Im$ is close to one.

Similarly, the stability plot of DPE is presented in Figs. 12a, 13, 14 and 15d. This simulation is done with different initial conditions and fractional-orders. It can be shown that both the human and vector trajectories tend towards the DPE over time provided ${\mathcal {R}}_0>1$, and this is in agreement with Theorem 4.3. Hence, the solution curves are stable and approach the DPE. However, the fractional-order plays a significant role in the manner the trajectories approach the DPE over time. It is important to note that, as fractional-order, $\Im$ gets near 1, the trajectories sufficiently gets close to the disease-present equilibrium and vice versa. Thus, it may be said epidemiologically that dengue and COVID-19 infections will persist within the population if ${\mathcal {R}}_0>1$ and $\Im$ substantially close to 1.

## Conclusion

In this paper, we have designed and analyzed a fractional-order mathematical model for the co-circulation of double strains of dengue and COVID-19 endowed with Caputo derivative. We established the existence and uniqueness of the solution for the given model through some fixed point theorem. The model’s solution was analyzed with the non-standard difference scheme. We also highlighted the impact of different Caputo fractional values on the dynamics of the diseases. Furthermore, we determined the impact of some parameters on the dynamics of the co-circulation. The local stability and global asymptotic stability of the disease free and endemic equilibria, were established using an appropriate Lyapunov function. This was validated via the phase portraits of the infected compartments.

Summary of main findings and implications to health officials.

(i) It was found that fractional-order has significant impact on the disease dynamics as demonstrated in Figure (). When the reproduction is less than unity, it was observed that as the fractional-order increases, all the human populations except ${R_{C}}$(COVID-19-recovered individuals) were decreasing. The implication is that, while effort is being made to lower the reproduction number, serious effort should be made also by the government and other related health organizations in understanding the impact of fractional-orders in the dynamics and control of dengue and COVID-19 infections. This is to ensure that the fractional-order is high as possible in order to reduce the viral load and increase the number of ${R_{D}}$ (dengue-recovered individuals). For the particular case of COVID-19-recovered individuals, efforts should be made to keep fractional-order as low possible to enhance the number of individuals recovering from COVID-19. Furthermore, in a situation where the reproduction is greater than unity, it is expected that the co-circulation of the infections will persist. However, a fractional-order as low as possible can substantially reduce the populations of COVID-19 related components while, a fractional-order as high as possible can significantly reduce the number of individuals infected with both strains of dengue virus. In any case, understanding the phenomenon of fractional-order as applied to disease dynamics will enhance the struggle of health officials in controlling dengue and COVID-19 infections.
(ii) It was shown that the disease reproduction numbers are functions of corresponding transmission and recovering rates as depicted in Fig. 11. It follows that an increasing transmission rate results to an increasing reproduction number and a decreasing recovery rate returns a decreasing reproduction number. Thus, to reduce the number of secondary infections caused by a typically infected person to below one, health officials should ensure that the transmission rate is reduced to barest minimum. This may be achieved by the use of treated nets, insecticides etc may reduce transmission rate of dengue virus. Similarly, the use of face-masks, lock-downs as observed during the epidemic can reduce the transmission rate of COVID-19. Furthermore, effort should be be made by health officials and governments to enhance recovery strategies and interventions. This could be possible through equipping the hospitals, sufficient supply of health workers and training etc.
(iii) It was observed that the disease-free equilibrium is globally asymptotically stable when ${\mathcal {R}}_0< 1$ while, the disease-present equilibrium is globally asymptotically stable when ${\mathcal {R}}>1$. This is demonstrated in Theorems 4.2 and 4.3 and validated with Figs. 12, 13, 14, 15, 16, 17, 18 and 19. Hence, to ensure a population free from co-circulation of double strains of dengue and COVID-19, health officials must strive to reduce the average secondary infections caused by one infected person to less than one. This may be achieved faster when the fractional-order is low. Unfortunately, the co-circulation will persist if average secondary infections produced by an infected is above one.

Our model is primary built on double strains of dengue and COVID-19 co-circulation. Furthermore, our model did not take into account the impact of vaccine for COVID-19 in the system. Future directions of our work could look at other more efficient numerical scheme for obtaining a solution, and other biological aspect of the model such as within-host dynamics.

## Data Availability

Data sharing is not applicable to this article as no new data was created or analyzed in this study.
